# Climate change and antimicrobial resistance in the Western Pacific: a mixed-methods systematic analysis

**DOI:** 10.1016/j.lanwpc.2025.101772

**Published:** 2025-12-16

**Authors:** Lianping Yang, Zishu Ma, Fanqian Meng, Ruonan Wang, Shanquan Chen, Chaojie Liu, Hung Chak Ho, Mingli Xu, Alvin Qijia Chua, Li Yang Hsu, Yanhui Jia, Yi Zhang, Cunrui Huang, John S. Ji

**Affiliations:** a School of Public Health, Sun Yat-sen University, Guangzhou, China; b Institute for Global Health and Development, Peking University, Beijing, China; c School of Public Health, University of Hong Kong, Hong Kong SAR, China; d School of Psychology and Public Health, La Trobe University, Melbourne, Australia; e Department of Public and International Affairs, City University of Hong Kong, Hong Kong SAR, China; f School of Politics and Public Administration, South China Normal University, Guangzhou, China; g Saw Swee Hock School of Public Health, National University of Singapore and National University Health System, Singapore; h Yong Loo Lin School of Medicine, National University of Singapore, Singapore; i Vanke School of Public Health, Tsinghua University, Beijing, China

**Keywords:** Climate change, Antimicrobial resistance, Climate-resilient health system, Vulnerability

## Abstract

**Background:**

Climate change and antimicrobial resistance (AMR) are escalating public health threats globally. The Western Pacific Region faces unique climatic and socioeconomic vulnerabilities, but evidence on this climate-AMR intersection is limited. We aimed to systematically provide evidence on this critical issue.

**Methods:**

We conducted a three-stage mixed-methods systematic analysis: (1) a narrative review mapping the regional AMR landscape and summarizing potential climate-driven mechanisms; (2) a systematic review (PubMed and Google Scholar, January 2000–March 2025) of regional quantitative studies; and (3) an empirical quantitative analysis using a longitudinal panel dataset. This analysis completes our systematic approach by visualizing AMR mortality trends (using data from the GRAM project) and applying regression analysis to model AMR-attributable death rates based on climatic and socioeconomic factors, providing quantitative evidence of the regional situation and its potential drivers.

**Findings:**

Literature review evidence showed that increasing temperature caused by climate change directly accelerates bacterial growth and resistance mutation rates and indirectly affects healthcare disruptions and antibiotic misuse during extreme weather events. We included 18 quantitative studies synthesised using the SWiM framework, which provided more specific evidence that higher temperatures are associated with increased clinical resistance rates and enhanced environmental dissemination of antibiotic resistance genes (ARGs). Our quantitative analysis found that a 1 °C increase in mean ambient temperature was associated with higher AMR-attributable mortality from carbapenem-resistant *Acinetobacter baumannii* (CRAB; β = 0.652, 95% CI 0.579–0.724, p < 0.001) and carbapenem-resistant *Pseudomonas aeruginosa* (CRPA; β = 0.422, 95% CI 0.304–0.541, p < 0.001). It also revealed that socioeconomic factors have heterogeneous effects.

**Interpretation:**

Climatic conditions and socioeconomic vulnerabilities jointly shape AMR risks in the Western Pacific Region. Projected increases in extreme weather events threaten to strain healthcare systems further and worsen antibiotic misuse. Strengthening climate-resilient health systems, improving multisectoral AMR governance, and establishing integrated AMR–climate surveillance networks are essential regional priorities.

**Funding:**

This work is supported by 10.13039/100004423World Health Organization (WPRO/2024-02/AGE-DHP/22552 4), 10.13039/501100001809National Natural Science Foundation of China (82422064, 82250610230, 72374228, 72074234), Natural Science Foundation of Beijing (IS23105), National Bureau for Disease Control and Prevention (20241660047), Guangzhou Basic and Applied Basic Research Program, China (2025A04J5118), Fundamental Scientific Research Funds for Central Universities, China (SYSU-25wkjc02), National Major Science and Technology Project of China (No. 2024ZD0524500), and Singapore National Medical Research Council (CoSTAR-HS CG21APR2005; AMRITS MOH-001326-01).

**Research in context**

**Evidence before this study**

We searched PubMed and Google Scholar (January 2000–March 2025). Search terms combined three domains: (1) climate exposures (e.g., “temperature”, “precipitation”), (2) AMR outcomes (e.g., “antimicrobial resistance”, “antibiotic resistance genes”), and (3) Western Pacific country identifiers. Most available studies were reviews and ecological analyses. These studies describe plausible pathways through which climate change may influence AMR, with several quantitative investigations suggesting that temperature and extreme weather events could contribute to increasing resistance. However, global syntheses rarely consider the specific context of the Western Pacific. Furthermore, the existing literature lacks a macro-level quantitative analysis that integrates these climatic pressures with the region's complex socioeconomic drivers. This significant research gap underscores the need for a region-specific, mixed-methods analysis to inform a tailored strategy.

**Added value of this study**

This study provides a three-stage analysis of the climate-AMR nexus in the Western Pacific. First, it maps the severe AMR burden, stark regional disparities, and systemic vulnerabilities (e.g., 50% inappropriate antibiotic use). It also revealed that both direct and indirect mechanisms through which climate change could accelerate AMR dissemination. We synthesized 18 regional studies that provided specific evidence linking climate change to AMR. Finally, our quantitative analysis found that a 1 °C temperature rise was significantly associated with higher CRAB mortality (β = 0.652, 95% CI 0.579–0.724, p < 0.001) and CRPA mortality (β = 0.422, 95% CI 0.304–0.541, p < 0.001). This analysis also identified strong and varied socioeconomic effects. Better governance measured by the Corruption Perceptions Index, showed a clear protective association with CRPA (β = −0.696, 95% CI −0.903 to −0.488, p < 0.001). To our knowledge, this is the first study to combine systematic evidence synthesis with region-wide quantitative analysis to examine climatic and socioeconomic correlates of AMR mortality in the Western Pacific.

**Implications of all the available evidence**

The combined evidence demonstrates that climate change interacts with socioeconomic and health-system vulnerabilities to exacerbate AMR risks in the Western Pacific. These findings confirm the insufficiency of uniform, single-sector interventions. This necessitates that AMR National Action Plans be implemented by empowered, multisectoral “One Health” task forces that integrate environmental and meteorological agencies. Furthermore, urgent investment in climate-resilient health infrastructure and the establishment of an integrated AMR–climate surveillance network is required. Future research must prioritise closing the profound evidence gap in Pacific Island Nations, developing causal inference models to disentangle complex pathways, and evaluating integrated interventions in real-world settings.

## Introduction

Climate change and antimicrobial resistance (AMR) are increasingly linked. For example, rising temperatures have been shown to accelerate bacterial growth, promote resistance mutations, and enhance the frequency of horizontal gene transfer (e.g., bacterial conjugation), resulting in higher resistance rates.^1^, ^2^, ^3^ Similarly, increased rainfall and the growing frequency of extreme weather events can damage sanitation and wastewater infrastructure, thereby enhancing the expression and spread of certain antibiotic resistance genes (ARGs) in the environment.^4^, ^5^, ^6^ As the primary treatment for infectious diseases, antimicrobial drugs face the challenge of AMR and a cascading increase in the disease burden.^7^ The UN General Assembly High-Level Meeting on AMR 2024 stressed that AMR will cause even more global suffering without decisive action, particularly in low- and middle-income countries. Recent estimates indicate that 4.71 million deaths in 2021 were associated with bacterial AMR, including 1.14 million that can be directly attributed to bacterial AMR, with the forecasts suggesting that associated deaths could reach 8.22 million and attributable deaths could rise to 1.91 million in 2050.^8^ Consequently, the intersection of climate change and AMR is receiving increasing attention. A recent scoping review shows the wide range of current research, finding that previous studies have focused on harmful synergies (39%), direct climate-driven AMR (19%), and shared solutions (12%).^9^

The Western Pacific region refers to one of the six regions of the World Health Organization (WHO). Headquartered in Manila, Philippines, the region comprises 38 countries, territories and areas, with a population of 1.9 billion people spanning high-income nations (e.g., Australia) and low-income countries (e.g., Papua New Guinea). The interplay of diverse climates, dense populations, and socioeconomic disparities creates unique vulnerabilities that demand targeted analysis. This is particularly relevant given that the region frequently faces environmental stressors, such as rising temperatures and sea levels, in addition to significant challenges related to AMR.^10^, ^11^, ^12^ A recent forecast predicts that South Asia, Southeast Asia, East Asia, Oceania, and sub-Saharan Africa will face the highest burden of AMR in the coming years.^8^ In many countries, weak healthcare infrastructure and low economic development make it even more difficult to control antimicrobial drug misuse and resistance.^13^ It is worth noting that as Pokharel et al. pointed out, AMR is not merely a regional problem but a matter of global equity, as its burdens disproportionately affect low- and middle-income countries. Therefore, addressing the issue requires special attention to these vulnerable regions, with a focus on developing targeted strategies and strengthening international cooperation to ensure more equitable progress.^14^^,^^15^ Without effective intervention, the Western Pacific region risks becoming one of the most severely affected areas.

Despite the mounting global evidence and the Western Pacific region's clear vulnerability, there is limited research focused on the intersection between AMR and climate change. A comprehensive analysis that synthesises the available regional evidence, analyses region-specific trends, and contextualizes these findings within the Western Pacific's unique climatic and socioeconomic landscape is notably absent. This systematic analysis addresses these gaps through a comprehensive analysis of climate-AMR interactions, integrating global evidence with a targeted focus on the Western Pacific's unique vulnerabilities. Ultimately, this study aims to contribute to the development of effective AMR policy and management strategies, particularly in the context of climate change adaptation and multi-sectoral governance.

## Methods

### Study design

This study was conducted in three stages to provide a comprehensive overview of the relationship between climate change and AMR in the Western Pacific Region. First, a narrative review was performed to characterize the regional AMR landscape and conceptual mechanisms. Second, a systematic review synthesized specific evidence on the climate-AMR link within the Western Pacific region. Finally, a quantitative analysis using a longitudinal panel dataset was undertaken to assess regional, macro-level associations between climatic, socioeconomic, health system factors and AMR.

### Stage 1: narrative review

A focused narrative review was conducted to map the current scientific understanding of mechanisms linking climate change and AMR and to identify key conceptual and empirical gaps. Purposive searches were performed in PubMed and Google Scholar (January 2000–March 2025) to identify review articles, mechanistic laboratory studies, and large-scale observational analyses relevant to biological, ecological, and transmission pathways. Selection prioritized papers that provided mechanistic insight or broad syntheses; these sources informed the conceptual framing and the selection of variables for subsequent quantitative analyses. To ensure a comprehensive overview, our search was complemented by a review of grey literature from WHO. Detailed search terms are provided in Supplementary Appendix A.

### Stage 2: systematic review

This systematic review component was not prospectively registered in PROSPERO because it was conducted as an integral part of a larger pre-planned mixed-methods study rather than as a stand-alone systematic review. The review protocol, including review questions, search strategy, eligibility criteria, and risk-of-bias assessment methods, was defined a priori and has been reported in full accordance with PRISMA 2020 and PRISMA-ScR guidelines (see Supplementary Appendix B). This approach ensures full transparency and reproducibility while maintaining the methodological coherence of the overall mixed-methods design. To synthesize primary, region-specific evidence, searches were performed in PubMed and Google Scholar (January 2000–March 2025) using combined keyword sets for (1) climate exposures (e.g., *temperature, precipitation, heatwave*), (2) AMR/ARG outcomes, and (3) Western Pacific country identifiers. The specific retrieval strategy can be found in Supplementary Appendix B.

Two reviewers independently screened titles/abstracts and full texts against pre-specified inclusion criteria: original quantitative studies (ecological, time-series, cross-sectional, cohort) or environmental metagenomic studies that reported extractable associations between climate variables and clinical AMR or environmental ARG metrics. We excluded reviews, commentaries, laboratory-only reports without field linkage, non-English publications and studies conducted outside the region. Study selection followed PRISMA 2020 procedures (see Supplementary Figure S1).

Due to high clinical, methodological, and statistical heterogeneity (e.g., variation in pathogens, climate metrics, study designs, and outcomes) across the included studies, a quantitative meta-analysis was deemed inappropriate. A Synthesis without Meta-analysis (SWiM) approach^16^ was adopted to systematically synthesize the evidence on the association between climate change and AMR in the Western Pacific region. Studies were grouped thematically (clinical/epidemiological, environmental, mechanistic), and results were summarized by effect direction and magnitude. The risk of bias (RoB) assessment, the full list of included studies and the synthesis of findings are provided in Supplementary Tables S1–S4.

### Stage 3: secondary data analysis and modelling

#### Data sources, variables, and visualization

**Fig. 1:**
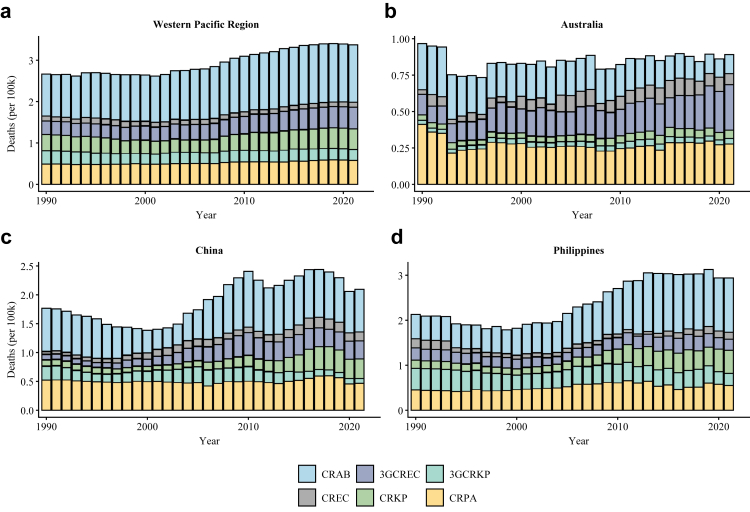
Trends of attributable death rates from six pathogen–antibiotic combinations across countries and regions in the Western Pacific. The panels show attributable death rates (per 100,000 population per year) for (a) the Western Pacific Region, (b) Australia, (c) China, and (d) the Philippines. The average for the Western Pacific Region (a) was calculated from 32 countries and regions with available data. Data were sourced from the Global Research on Antimicrobial Resistance (GRAM) project. Pathogen-antibiotic combinations are: CRAB = carbapenem-resistant *Acinetobacter baumannii*; CRPA = carbapenem-resistant *Pseudomonas aeruginosa*; 3GCRKP = third-generation cephalosporin-resistant *Klebsiella pneumoniae*; CRKP = carbapenem-resistant *Klebsiella pneumoniae*; 3GCREC = third-generation cephalosporin-resistant *Escherichia coli*; CREC = carbapenem-resistant *Escherichia coli*.

To provide quantitative evidence for our review, we extracted country-level AMR mortality estimates (1990–2021) for six WHO-priority pathogen groups from the Global Research on Antimicrobial Resistance (GRAM) project, which is conducted by the Institute for Health Metrics and Evaluation (IHME) at the University of Washington (https://vizhub.healthdata.org/microbe/). GRAM provides standardized estimates of AMR burden using data from surveillance systems, systematic reviews, and national health datasets. We calculated and visualized average standardized death rates (per 100,000 population per year) for the Western Pacific Region and for three representative countries—China, Australia, and the Philippines—chosen for their distinct socioeconomic and climatic profiles (Fig. 1).

Using the GRAM mortality estimates as outcome data, we constructed a longitudinal panel dataset integrating climate, socioeconomic, and health system variables from the Climatic Research Unit Time series and the World Bank. Predictor variables included mean ambient temperature (Tmp), precipitation (log_PREC), population density (log_PD), hospital beds (log_HD), health expenditure (HEG), urban population (UP), corruption perceptions index (CPI), antimicrobial consumption (AMC), and access to safe water and sanitation (WASH). The final analytical sample comprised 13 countries over 23 years (1999–2021), yielding 299 country-year observations. A full description of data sources, variable definitions, and descriptive statistics is provided in Supplementary Tables S5 and S6.

#### Statistical modelling

We applied a series of multivariable linear regression models to examine the associations between climate and socioeconomic factors and AMR-attributable mortality. The dependent variable was the log-transformed AMR-attributable death rate (per 100,000 population per year). Separate models were run for each of the six priority pathogen groups (carbapenem-resistant *Acinetobacter baumannii* (CRAB), carbapenem-resistant *E. Coli* (CREC), carbapenem-resistant *Klebsiella pneumoniae* (CRKP), carbapenem-resistant *Pseudomonas aeruginosa* (CRPA), third-generation cephalosporin-resistant *E. Coli* (3GCREC), and third-generation cephalosporin-resistant *K. pneumoniae* (3GCRKP)). All models used pooled ordinary least squares (OLS) estimation with robust standard errors to ensure valid inference. Fixed-effects panel models were also tested, but they produced unstable coefficients, likely due to limited within-country variability in climate and health system indicators. Therefore, pooled OLS results are presented as the primary findings, with fixed-effects results provided in the Supplementary S8–S11.

All statistical analyses were conducted in R (v4.5.1).

### Ethics statement

The ethical approval was not required for this study. The research employed a mixed-methods approach, consisting of a review of published literature and quantitative data analysis. The analysis relied on publicly available data, with no personally identifiable information included.

### Role of the funding source

The funders had no role in the study design, data collection, analysis, interpretation, or writing of the report.

## Results

### Epidemic trend of AMR in the Western Pacific Region

In the Western Pacific region, home to more than one quarter of the world's population, AMR presents a growing challenge influenced by regional factors. This phenomenon has caused significant health and economic impacts. For example, AMR is rendering antibiotics ineffective for treating common infections and slowing the process of the control and elimination of high-risk infections such as malaria, sexually transmitted infections and Tuberculosis (TB). According to the WHO estimates, nearly 90,000 cases of multidrug-resistant TB were estimated to have occurred in the Western Pacific region in 2017, yet only 30% of them were diagnosed.^17^ In a worst-case scenario, the WHO projects that AMR could have caused 450,000 deaths in 2020, corresponding to a mortality rate of 23.5 per 100,000 population. If current trends continue, the cumulative number of AMR-related deaths in the region is forecast to reach 5.2 million from 2020 to 2030, with a total economic cost approaching $150 billion, which is higher than Australia's total health expenditure in 2019 (US$ 136.8 billion).^18^^,^^19^

Data from the Global Research on Antimicrobial Resistance (GRAM) project further highlights the growing AMR challenge in the Western Pacific region. From 1990 to 2021, the region experienced a clear upward trend in attributable mortality rates. Carbapenem-resistant pathogens, particularly *A. baumannii* (CRAB), shows the most significant increase in death rates.

This general upward trend, however, obscures substantial intraregional variations. As shown in Fig. 1, countries with divergent economic and healthcare levels exhibit vastly different death rates. Notably, as the region's largest developed country, Australia has successfully stabilized or even reduced the burden of several key resistant pathogens, notably showing a significant decline in deaths rate attributable to CRAB. This stands in sharp contrast to the situation in China and the Philippines, where the death rates from CRAB and most other priority pathogen-antibiotic combinations continues to rise. Two studies on the prevalence of CRAB also emphasized this difference. These studies found that Japan (2.8%) and Australia (6.5%) report the lowest rates, likely benefiting from robust infection control measures. In contrast, many neighbouring low- and middle-income countries have reported resistance proportions exceeding 80%.^20^^,^^21^

Several studies have also underscored the severe circumstances in low- and middle-income countries. A retrospective study in China shows that resistance rates among clinical isolates have increased markedly, particularly for *Escherichia coli* and *Staphylococcus aureus*.^22^ The Philippines, an economically underdeveloped island country in the region, demonstrated a steady increase in resistance rates for several key pathogen-antibiotic combinations during 2010–2020, including carbapenem-resistant organisms.^23^ Similarly, in Pacific island countries, where data is limited, available evidence indicates significant drug resistance threats, with methicillin-resistant *S. aureus* (MRSA) incidence varying markedly, from over 50% in Papua New Guinea to under 20% in other territories.^24^ However, the interpretation of these latter results needs to be cautious, as they may be influenced by insufficient diagnostic capacity.^12^

### Socioeconomic drivers of AMR in the Western Pacific

The severe situation and continuous rise of AMR in the Western Pacific are driven by a combination of powerful drivers. A primary factor is the widespread overuse and misuse of antimicrobials. The WHO states that the overuse and misuse of antibiotics continue to be a serious problem in the region, with around 50% of antibiotic consumption considered inappropriate or irrational.^25^ And behind this phenomenon is the result of multiple intersected factors. For example, the loose regulations that permit easy over-the-counter access to antibiotics without a prescription, which is still a serious problem in many parts of the region.^26^ It is also deeply impacted by the socio-cultural norms, with self-medication and a dependence on antibiotics being common practices.^27^, ^28^, ^29^ Inadequate laboratory and diagnostic capacity is another key bottleneck, especially for Pacific island countries. It often leads the clinicians rely on empirical treatment with broad-spectrum antibiotics.^30^ Inadequate infection prevention and control measures in healthcare settings, coupled with poor sanitation and hygiene in communities, accelerate the spread of resistant bacteria. In addition, The weak infrastructure also makes waste water and wastewater treatment plants appeared to serve as the main sources for the development and spread of antibiotic resistance in the Western Pacific region.^31^ Urbanization and population density in cities like Manila exacerbate these issues, as they contribute to heightened transmission risks.^17^ There is also evidence suggesting a correlation between a low density of physicians and increased rates of drug resistance,^32^ though the specific mechanistic evidence for this in the Western Pacific region remains insufficient.

### Global context: how climate change shapes AMR trends

Some existing ecological studies have found that the rising temperatures and changing precipitation patterns are associated with the prevalence of AMR.^33^ Multiple studies conducted in the Americas, Europe, and China have shown that increasing environmental temperatures exacerbate antibiotic resistance rates in populations.^34^, ^35^, ^36^, ^37^ However, research has yet to definitively clarify the relationship between other climate change factors, such as precipitation and humidity, and the prevalence of AMR at the population level. One recent study on quinolone-resistant *E. coli (QREC)*, for instance, found higher resistance rates in warmer monsoon regions, while lower resistance rates were observed in cooler mountainous climates and areas with higher rainfall.^38^ Moreover, climate change's impact on AMR extends to the environmental presence and dissemination of ARGs, which are the fundamental drivers of bacterial resistance.^39^ ARGs are ubiquitous in natural environments such as water bodies and soils, which serve as reservoirs for these genes.^5^ Climate changes, particularly temperature and precipitation, may exacerbate the abundance of these genes.^40^, ^41^, ^42^

**Fig. 2:**
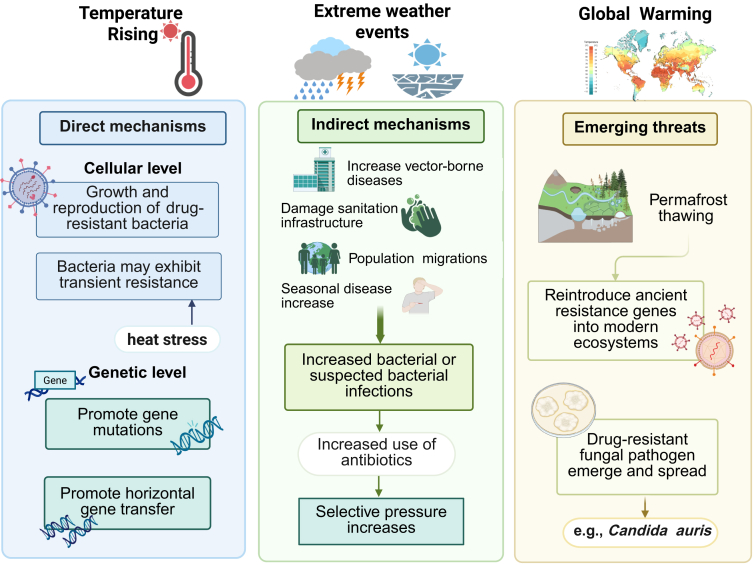
The potential mechanism of climate change driving AMR. The figure illustrates three primary pathways. The direct mechanisms (left panel) include cellular-level impacts from rising temperatures (e.g., accelerated bacterial growth) and genetic-level impacts (e.g., promotion of gene mutations and horizontal gene transfer). The indirect mechanisms (middle panel) show how extreme weather events can damage sanitation infrastructure, increase bacterial infections, and drive antibiotic misuse, leading to increased selective pressure. The emerging threats (right panel) include the reintroduction of ancient resistance genes from thawing permafrost and the emergence of drug-resistant fungal pathogens.

Various climate-related factors can influence AMR through multiple mechanisms, ranging from the cellular to the genetic level (Fig. 2). To elucidate, rising temperatures can promote bacterial growth and reproduction, potentially increasing the likelihood of their transmission.^43^ Moreover, under heat stress conditions, bacteria may exhibit transient resistance as a self-protection mechanism.^44^ While this surface-level response is typically short-lived, prolonged or continuous heat stress can have deeper impacts on bacterial genetic material.^6^ At the genetic level, climate change (notably temperature increases) can enhance AMR by promoting gene mutations and horizontal gene transfer.^45^ For example, overuse of antibiotics or temperature changes may cause stress responses in cells, leading to persistent or adaptive evolution, ultimately resulting in genetic mutations that lead to AMR.^40^^,^^46^ Horizontal gene transfer is another pathway for bacteria to acquire resistance besides mutations, and it also occurs at the genetic level.^1^ Research has shown that climate change, such as rising temperatures or increased rainfall, may accelerate this process.^47^^,^^48^

In addition to its direct impacts, other climate-related phenomena can indirectly influence AMR through extreme weather events, such as high temperatures, droughts, or floods, and their associated socio-economic consequences. For instance, higher temperatures may increase the spread of vector-borne diseases and raise the risk of infections within healthcare systems.^49^ Flooding and other extreme weather events, which often cause water-related disasters, can contaminate water supplies and damage sanitation infrastructure, increasing the risk of waterborne diseases.^50^ Drought-induced shortages of water and food may trigger large-scale population migrations, heightening the risk of bacterial transmission and infection within and between population.^51^ An increase in bacterial infections is likely to drive the overuse or misuse of antibiotics. Furthermore, global warming is associated with a higher incidence of febrile illnesses (such as influenza, acute bronchitis, and pneumonia) that may have seasonal patterns.^52^ Due to the often unclear etiology of these conditions, they are commonly managed with empirical antibiotic therapy. The increase in antibiotic use is linked to greater selective pressure on bacterial populations.^53^ Selective pressure, which refers to the exposure of bacteria to an environment where susceptible strains are eliminated while resistant strains survive and proliferate, exemplifies the principle of “survival of the fittest”.^54^ As the frequency and scope of antimicrobial use increase, the likelihood of selecting for resistant bacteria also rises, accelerating the spread of AMR.^55^

Furthermore, climate change provides new sources for the emergence and spread of AMR. Global warming and permafrost thawing may reintroduce ancient resistance genes into modern ecosystems, providing new sources for the emergence and spread of AMR.^56^^,^^57^ In addition, studies have found that the emergence and spread of the highly drug-resistant fungal pathogen *Candida auris* could be linked to global climate change.^58^ This pathogen, often characterized by multidrug resistance, poses a significant threat to healthcare institutions worldwide.^59^

### Particularity of geography and climate change in the Western Pacific Region

The Western Pacific region's geography is characterized by densely populated urban centres, vast oceanic areas, and numerous islands. This diversity is further complicated by the region's climate, which ranges from tropical to temperate, with significant variations in temperature and precipitation patterns. The WHO highlights that these geographical particularities make the region particularly vulnerable to the impacts of climate change. For instance, sea-level rise (SLR) and increasing storm intensity pose significant risks to island nations, affecting water supply and sanitation systems, which are critical components in controlling the spread of infectious diseases and resistant pathogens.^60^ SLR also poses a significant threats for populations living in low-lying areas at or near coastlines, which may lead to the uncertain population mobility.^61^ Additionally, more frequent natural disasters associated with climate change add to the region's disease burden. Globally, eight of the countries most prone to natural disasters are in the Western Pacific region. Both floods (more common in Asian countries) and cyclones and storms (to which island countries are particularly prone) have increased in frequency and severity in recent decades, which now results in 8.7 million people being internally displaced in the region every year.^17^ The unique geographical and climatic conditions create a complex interplay of factors that can exacerbate the emergence and spread of resistant pathogens.

### Synthesis without meta-analysis (SWiM): the Climate-AMR nexus in the Western Pacific Region

A total of 18 primary studies were included in this SWiM. The studies predominantly concentrated on China (N = 14), with additional investigations in Korea (N = 2), Japan (N = 1) and Australia (N = 1), providing a strong regional focus. Study characteristics are summarized in (Supplementary Table S3).

The included studies were categorized into three groups based on the One Health framework to construct an evidence chain: **A. Clinical/Epidemiological:** 5 studies focused on statistical associations between climate factors and clinical AMR rates; **B. Environmental:** 9 studies examined the abundance, diversity, and spread of ARGs in various natural and engineered environments; **C. Mechanistic:** 4 real-data-based studies investigated the molecular or cellular mechanisms by which temperature or other environmental factors directly influence resistance development or transfer.

The synthesis is structured to follow the logical flow of the One Health concept, from the clinical burden to environmental reservoirs and the underlying biological mechanisms.

#### A. Clinical and epidemiological evidence

An analysis of Chinese nationwide panel data (2005–2019) demonstrated that a 1 °C increase in average ambient temperature was associated with a 1.14-fold increase in CRKP prevalence and 1.06-fold increase in CRPA prevalence.^36^ Two studies utilizing the DiD approach found that every 1 °C increase in annual average temperature was associated with a 4.7% increase in 3GCRKP (RR: 1.047, 95% CI 1.031–1.082) and a 10.7% increase in CRKP,^62^ while every 1 °C increase in average ambient temperature was associated with an increase of 2.71% in 3GCREC, 32.92% in CREC, and 1.81% in QREC.^63^ The positive correlation was also identified for QREC across 31 Chinese provinces. Core findings revealed significant regional and climatic variations in QREC resistance, with higher rates in warmer climates.^38^ Furthermore, the resistance rate of erythromycin-resistant *Streptococcus pneumoniae* (ERSP) was shown to be influenced by complex geographical, demographic, and climatic factors, highlighting the multifaceted nature of the epidemiological link.^64^

#### B. Environmental and reservoirs evidence

Elevated temperature was a main factor driving the environmental AMR. In the Yellow River, metagenomic analysis indicated that gradually raised temperature could reduce overall ARG diversity but significantly increase the total abundance of ARGs, particularly multidrug-resistance and high-risk ARGs.^42^ Similarly, in natural soil ecosystems, climate warming increased the proportions of specific ARGs, with the effect being highly dependent on seasonality.^41^ In constructed wetlands, Temperature was the main driving factor of ARGs and mobile genetic elements (MGEs), and ARG abundance was highest in summer.^65^

Rainfall events were also identified as a critical mechanism for ARG spread. Studies in China demonstrated that rainfall facilitates the transmission and proliferation of ARGs from ambient air to soil.^47^ Hydrometeorological influences and rainfall events, often linked to combined sewage overflows (CSOs) or sewage contamination, led to elevated levels of ARGs and enteric bacteria in seawater in Australia^66^ and Korea.^67^

In Tibetan glaciers, ARG abundance was found to be higher in monsoon-influenced glaciers than the westerly-dominated glaciers, potentially linked to higher antibiotic usage in the Indian subcontinent.^68^ Another study of Tibetan Plateau Wetlands showed that the warming climate trend will very likely lead to increased soil temperature and moisture, which will alter the microbial community composition and increase the background levels of ARGs.^69^ Estuarine systems in China showed that climate variables explained 44% of ARG variance, with temperature as the most important climate driver.^70^

#### C. Mechanistic and in vitro evidence

An *in vitro* study demonstrated that the transmission and stability of carbapenemase-encoding plasmids in *K. pneumoniae* are highly temperature-dependent, with distinct optimal temperatures.^71^ In *A. baumannii*, meropenem resistance rates peaked in colder months. Resistant strains exhibited greater resilience to low-temperature (4 °C) stress by upregulating key resistance-related genes, suggesting a survival advantage for resistant strains in colder periods.^72^ In the Japanese lake environment, antibiotic resistance in *E. coli* is more likely to be induced at temperatures between 25 and 37 °C, particularly in tropical regions with longer exposure times.^73^ In marine settings, *Vibrio harveyi* from warmer South China Sea waters harboured more virulence genes and greater drug resistance, suggesting climate-AMR-aquaculture linkages.^74^

Supplementary Table S4 summarizes the synthesis of findings (SWiM) on the Climate-AMR Nexus in the Western Pacific Region. The synthesis highlights that elevated ambient temperature is consistently associated with increased clinical resistance rates for pathogens such as CRAB, CRKP and QREC. Both increasing environmental temperature (water/soil) and hydrometeorological factors (rainfall/monsoon) are consistently identified as major drivers of ARG distribution and abundance. Elevated temperature is linked to the increased abundance of high-risk ARGs, while rainfall/runoff facilitates the transmission and proliferation of ARGs from sources (e.g., ambient air, soil) into receiving water bodies. Experimental evidence also showed that warmer temperatures significantly enhance the frequency of horizontal gene transfer, providing biological plausibility for the clinical association.

### Regional quantitative analysis: climate and socioeconomic determinants of AMR mortality

Descriptive statistics for key climatic, socioeconomic, and health system variables across 299 country-year observations (1999–2021) in the Western Pacific Region are summarized in Supplementary Table S6. The mean ambient temperature was 21.36 °C (SD 7.10), and the average annual precipitation reached 1560.26 mm (IQR 665.25–2019.45), reflecting substantial climatic heterogeneity across tropical and temperate zones. The average population-weighted PM_2_._5_ concentration was 19.16 μg/m^3^ (SD 10.22). Socioeconomic indicators showed wide heterogeneity, including health expenditure (mean 5.19%, SD 2.69), population density (median 129.97 people/km,^2^ IQR 67.75–344.93) and corruption perceptions index (mean score 50.70, SD 24.96). Precipitation (PREC), PM_2_._5_ (PMP), population density (PD), and hospital beds (HD) were logarithmically transformed in the regression analyses to reduce skewness and improve model stability. Bivariate correlation analyses (Supplementary Figures S2–S7) revealed moderate to strong correlations between climatic factors (temperature, precipitation, particulate pollution) and between health system and socioeconomic indicators (e.g., urban population, health expenditure, WASH). These correlations suggest that both climatic and socioeconomic factors may jointly influence AMR dynamics in the region.

We next examined how climate and socioeconomic factors were associated with AMR-attributable death rates across Western Pacific countries, using log-transformed mortality (per 100,000 population) as the dependent variable. Results from the six pathogen-specific models are presented in Table 1 (three best-fitting models: CRAB, CRPA, 3GCRKP) and Supplementary Table S7 (remaining models: CREC, CRKP, 3GCREC).

**Table 1:** Regression results of CRAB, CRPA and 3GCRKP.

	CRAB		CRPA		3GCRKP	
	β [95% CI]	p-value	β [95% CI]	p-value	β [95% CI]	p-value
Tmp	0.652 [0.579, 0.724]	<0.001	0.422 [0.304, 0.541]	<0.001	−0.047 [−0.132, 0.038]	0.277
log_PREC	0.140 [0.094, 0.186]	<0.001	0.110 [0.030, 0.190]	0.007	0.048 [−0.015, 0.110]	0.133
log_PM2.5	0.432 [0.316, 0.549]	<0.001	0.397 [0.196, 0.599]	<0.001	0.368 [0.244, 0.491]	<0.001
log_PD	−0.066 [−0.155, 0.022]	0.140	0.211 [0.075, 0.346]	0.002	0.169 [0.084, 0.255]	<0.001
log_HD	0.178 [0.066, 0.291]	0.002	0.146 [0.016, 0.276]	0.028	−0.607 [−0.723, −0.491]	<0.001
HEG	−0.072 [−0.156, 0.012]	0.091	0.825 [0.709, 0.941]	<0.001	0.081 [0.023, 0.139]	0.006
UP	−0.011 [−0.135, 0.114]	0.868	0.595 [0.371, 0.818]	<0.001	−0.400 [−0.541, −0.259]	<0.001
CPI	−0.245 [−0.347, −0.143]	<0.001	−0.696 [−0.903, −0.488]	<0.001	0.129 [0.015, 0.244]	0.027
AMC	−0.032 [−0.090, 0.026]	0.285	−0.196 [−0.283, −0.109]	<0.001	−0.123 [−0.198, −0.048]	0.001
WASH	0.113 [0.046, 0.179]	0.001	0.439 [0.349, 0.529]	<0.001	−0.273 [−0.347, −0.199]	<0.001
R2	0.86		0.69		0.85	
adj_R2	0.85		0.68		0.85	
N	299		299		299	

Climatic factors emerged as strong correlates of AMR-attributable death rate. Specifically, a 1 °C rise in mean ambient temperature was significantly associated with higher death rate for CRAB (β = 0.652, 95% CI 0.579–0.724, p < 0.001) and CRPA (β = 0.422, 95% CI 0.304–0.541, p < 0.001), whereas no significant association was observed for 3GCRKP (β = −0.047, 95% CI −0.132 to 0.038, p = 0.277). Similarly, greater precipitation was associated with increased mortality for CRAB (β = 0.140, 95% CI 0.094–0.186, p < 0.001) and CRPA (β = 0.110, 95% CI 0.030–0.190, p = 0.007). Higher population-weighted PM_2_._5_ concentrations were similarly associated with increased mortality, including for CRAB (β = 0.432, 95% CI 0.316–0.549; p < 0.001) and CRPA (β = 0.397, 95% CI 0.196–0.599, p < 0.001).

Socioeconomic and health system indicators showed heterogeneous effects across pathogens. The Corruption Perceptions Index (CPI) was inversely associated with AMR mortality, particularly for CRPA (β = −0.696, 95% CI −0.903 to −0.488, p < 0.001). The association of hospital bed density (log-HD) differed by pathogen, showing positive associations for CRAB (β = 0.178, 95% CI 0.066–0.291, p = 0.002) and CRPA (β = 0.146, 95% CI 0.016–0.276, p = 0.028), but was negatively association with 3GCRKP (β = −0.607, 95% CI −0.723 to −0.491, p < 0.001). Other socioeconomic variables—including health expenditure (HEG), urban population (UP), and WASH coverage—also exhibited significant but directionally variable associations across pathogens (Table 1).

Collectively, these findings suggest that the magnitude and direction of climatic and socioeconomic influences on AMR mortality differ substantially by bacterial species.

## Discussion

Our study indicated that despite mounting evidence that climate change impacts the occurrence and spread of AMR, the detailed mechanisms remain insufficiently understood. In the Western Pacific in particular, few studies have combined environmental data with resistance-surveillance trends, and relatively little attention is given to the integration of socio-economic factors, resulting a critical evidence gap.^9^ By combining systematic review findings with regional statistical analysis, we found that temperature rise and precipitation were generally associated with increased AMR mortality. However, these climatic effects are intertwined with complex socioeconomic conditions—such as healthcare capacity, governance quality, and population density, which may either amplify or mitigate their influence. This limited understanding hinders targeted policy formulation and development, making it difficult for policymakers to obtain the necessary evidence and recommendations to effectively address the dual threats of climate change and AMR. Bridging this gap requires using longitudinal, cross-sectoral data and integrating climate-science expertise to comprehensively investigate the climatic and socioeconomic contributions to AMR.

Despite the urgent need to link AMR trends with climate data, AMR surveillance across the Western Pacific region remains patchy. According to the 2023 WHO Tracking AMR Country Self-assessment Survey (www.amrcountryprogress.org), 21 countries in the Western Pacific region have developed and are implementing a National AMR Action Plan. However, few countries have revised their national AMR action plan or developed new ones when existing plans are set to expire (e.g., Japan, Australia, Mongolia). Similarly, most countries have not established a monitoring and evaluation plan for the national AMR action plan (e.g, Australia, New Zealand, Philippines, Fiji), which are all Island countries. While 7 countries regularly collect cross-sectoral AMR data to inform their national AMR action plan (Malaysia, Japan, China, Republic of Korea, Singapore, Tonga and Figi), leaving most countries with insufficient or under-investigated data collection. To fill this gap, we propose establishing a regional AMR–climate monitoring and evaluation network modelled on WHO's GLASS. The uniqueness of this network lies in its use of the intersection of AMR and climate change as the core monitoring content. This network should integrate diverse data streams including climate variables and environmental pollution data to build a crucial bridge between environmental and clinical discoveries. By utilizing big data analysis, the network will be able to track real-time trends, identify hotspots, and compile regular regional assessment reports.^18^^,^^75^^,^^76^

However, improving data systems alone is insufficient. Mitigating AMR under a changing climate demands robust multi-sector governance.^77^ AMR is a typical One Health issue, hence the governance of AMR highly requires the approach of One Health.^78^ The WHO, together with other Quadripartite organizations, namely the Food and Agriculture Organization of the United Nations, the United Nations Environment Programme and the World Organization for Animal Health, have called for a multisectoral One Health response, recognizing the significant impacts of AMR on human and animal health, food production and the environment, and emphasizing the need for collaboration, communication, and coordination across relevant sectors.^79^ Future research could focus more on the intersection between AMR and environmental factors, human behaviours, applying the approach of One Health. Many countries have established national action plans, but these action plans suffer from insufficient participation from the environmental department.^80^ National action plans to address AMR within the context of climate change must be effectively implemented through enhanced multi-sectoral governance mechanisms.^81^ This involves setting up dedicated agencies or task forces with clear roles to coordinate across health, environment, agriculture, livestock, meteorology, and education sectors. Comprehensive strategic planning should encompass antibiotic stewardship (e.g., WHO AWaRe classification), environmental pollution control, climate change adaptation, and public education initiatives. The integration of these strategies can enhance overall effectiveness.

It is worth noting that the socioeconomic landscape and distinctive climate of the region add complexity to AMR management. Economic vulnerability limits investment in comprehensive AMR and climate change strategies. Populations in low-income areas often face barriers to accessing quality healthcare, technical facilities and awareness, leading to reliance on over-the-counter antibiotics, which contributes to misuse and increased resistance.^82^ Socioeconomic disparities affect policy implementation: wealthier countries can afford stricter AMR controls and climate adaptation plans, while poorer nations struggle with resource allocation and prioritize immediate public health needs.^83^ Therefore, in the global process of addressing AMR, special attention must be paid to the special challenges faced by these low-and middle-income countries, and specific measures must be taken to promote fair and coordinated development.

In addition, the frequent occurrence of extreme weather events will further disrupt the functionality of health systems and infrastructure. Typhoons, floods, and droughts are among the frequent disasters that can cause damage to medical facilities, increase the demand for medical resources, and impact the stability of the pharmaceutical supply chain. Due to the extensive use of antibiotics in disaster response, these climate events may lead to their irrational use, further intensifying the issue of drug resistance. Climate change will exacerbate the demand for healthcare, straining healthcare systems in their efforts to address AMR and other climate-related health issues. The shortage of resources will impact the implementation of diagnostic, treatment, and prevention measures, thereby weakening the region's overall capacity to respond to AMR. A low proportion of medical staff may lead to more serious inappropriate prescriptions and self-medication.^84^ Previous studies have found that the shortage of nursing human resources may be accompanied by an increase in drug-resistant prevalence. And one potential factor is a shortage of nurses, which results in lower compliance with infection control measures.^85^

Given the impact of these dual challenges and prevailing socioeconomic constraints on the healthcare system, the Western Pacific region needs to strengthen the health system's resilience and establish a climate-adaptive healthcare management system.^86^ The health system's resilience refers to its ability to recover from challenges, regardless of whether climate change, health crises, or other disruptions cause them.^87^ Low resilience implies insufficient capacity to effectively manage climate-related health crises, particularly during emergencies such as floods or typhoons, potentially worsening AMR issues. Additionally, the enforcement of AMR-related regulations is generally weak. Measures to control antibiotic prescriptions and promote rational antimicrobial use are often ineffective.^88^ Therefore, aligning with WHO guidance, building climate-adapted infrastructure, embedding AMR protocols into emergency plans, and enhancing workforce training are essential to optimize resource use, minimize environmental release of ARGs and sustain care delivery amid increasingly unstable climate conditions.^89^^,^^90^

At the regional level, differences in policies and resources among countries hinder unified action and cross-border cooperation.^80^ Limited international collaboration results in inadequate regional resource sharing and consistent governance. Collaborative efforts should focus on multiple dimensions, including funding, innovation, and capacity building. Establishing a regional funding pool can support AMR initiatives and climate resilience projects across countries. For instance, the Western Pacific region could benefit from a shared fund that supports research and the implementation of joint projects to address AMR and climate change simultaneously. Moreover, supporting regional research institutions in developing new technologies, antibiotics, and monitoring tools will be critical.^91^

Ultimately, all these efforts are closely linked to achieving the Sustainable Development Goals (SDGs). AMR is a long-term threat to achieving universal health coverage (SDG 3) and ensuring population security.^92^ By strengthening AMR monitoring, multi-sectoral governance, health system resilience, and regional cooperation, we can not only better respond to public health crises but also promote environmental sustainability (SDG 13) and reduce inequality (SDG 10).^93^ This will contribute to a more equitable, healthier, and resilient future in the Western Pacific region and globally.

Our review has several limitations. First, the narrative review component is susceptible to certain selection and interpretation biases. Furthermore, while the death rate data we extracted from the GRAM project provides a valuable quantitative overview, it is based on estimation models and should be interpreted with caution due to potential inherent biases or inaccuracies compared to actual mortality data. Second, the existing body of research is geographically imbalanced. The current research mainly focuses on developed countries such as Europe and America, as well as China. There is a notable lack of comprehensive research that combines climate data with AMR data in low-income countries throughout the Western Pacific region, with a particular scarcity of studies focusing on Pacific Island Countries. Although existing studies provide evidence of associations between climate factors and AMR, the nature of this relationship remains insufficiently explored. These observed associations may reflect: (i) true climatic causality; (ii) spurious correlations driven by shared underlying drivers (e.g., weak infrastructure); or (iii) a combination of both. Additionally, the disease burden AMR poses under climate change remains challenging to estimate accurately. These research gaps hinder a deeper understanding of how climate change affects bacterial resistance, limiting the development of targeted strategies and interventions.

Future research should be supported to enhance our understanding of the nature of the causal relationship between climate change and AMR, especially in regions with high climate variability and AMR prevalence. Such research includes but are not limited to conducting in-depth mechanistic studies to elucidate how specific climate variables influence resistance transmission and development pathways. Disentangling these pathways requires causal inference frameworks and methods that explicitly integrate socioeconomic and health system data. Cross-regional and interdisciplinary collaboration is essential, integrating efforts from different countries and academic fields to promote holistic response strategies. Moreover, quantitative assessments of the AMR disease burden in the context of climate change should be strengthened to better estimate potential public health and economic impacts, providing stronger data support for policymakers.

### Conclusion

Our study maps the escalating threats of AMR and climate change, with a novel focus on the Western Pacific's unique vulnerabilities. Our findings show that the Western Pacific's diverse climates and dense populations amplify both direct mechanisms, such as temperature-driven bacterial growth, and indirect pressures, like antibiotic misuse during climate-triggered disasters. Our systematic review further identified specific regional associations, such as temperature-dependent resistance trends in clinical pathogens. Crucially, our quantitative analysis concludes that these climatic impacts are not uniform. They are profoundly mediated by socioeconomic factors, which have complex, heterogeneous effects on AMR death rate. This interaction underscores the insufficiency of single-sector AMR interventions. With over 5 million projected AMR-related deaths and US$150 billion in economic losses by 2030, the stakes for this region are alarmingly high. To tackle these risks, we propose a framework to include real-time surveillance to predict resistance spikes during typhoons and monsoons, multisectoral governance linking health, environment, and agriculture, climate-resilient health systems with strict antimicrobial stewardship, and regional collaboration through shared funding and data exchange.

## Contributors

Lianping Yang, John S. Ji, Zishu Ma, and Fanqian Meng conceived the study. Lianping Yang designed the methods and analysis framework. Lianping Yang, Zishu Ma, and Fanqian Meng searched, screened and reviewed the policy documents. Lianping Yang, Zishu Ma, Fanqian Meng extracted data from literature. Lianping Yang conducted statistical analyses. Lianping Yang, Zishu Ma, Fanqian Meng and John S. Ji led the writing processes. Ruonan Wang, Shanquan Chen, Chaojie Liu, Hung Chak Ho, Mingli Xu, Yanhui Jia, Yi Zhang, Alvin Qijia Chua, Li Yang Hsu and Cunrui Huang contributed to finding interpretation and draft revision. Lianping Yang and John S. Ji verified the data and had full access to the raw data. Authors approved the final version of the manuscript. John S. Ji had final responsibility for the decision to submit for publication.

## Data sharing statement

The data used in this study are extracted from published articles, reviews and reports as indicated in the main text and references. AMR mortality estimates (1990–2021) from the Global Research on Antimicrobial Resistance (GRAM) project, which is conducted by the Institute for Health Metrics and Evaluation (IHME) at the University of Washington (https://vizhub.healthdata.org/microbe/). Other climate, demographic, and socioeconomic variables were extracted from publicly accessible datasets. A comprehensive list of all data sources is provided in the Supplementary Material (Supplementary Table S5).

## Editor note

The Lancet Group takes a neutral position with respect to territorial claims in published maps and institutional affiliations.

## Declaration of interests

We declare no competing interests.

## Appendix A Supplementary data

Supplementary Material

Abstract in Chinese

## References

[1] Rzymski P., Gwenzi W., Poniedziałek B., Mangul S., Fal A. (2024). Climate warming, environmental degradation and pollution as drivers of antibiotic resistance. Environ Pollut.

[2] Rodríguez-Verdugo A., Lozano-Huntelman N., Cruz-Loya M., Savage V., Yeh P. (2020). Compounding effects of climate warming and antibiotic resistance. iScience.

[3] Zhao W., Zhang B., Zheng S., Yan W., Yu X., Ye C. (2025). High temperatures promote antibiotic resistance genes conjugative transfer under residual chlorine: mechanisms and risks. J Hazard Mater.

[4] Di Cesare A., Eckert E.M., Rogora M., Corno G. (2017). Rainfall increases the abundance of antibiotic resistance genes within a riverine microbial community. Environ Pollut.

[5] Larsson D., Flach C.-F. (2022). Antibiotic resistance in the environment. Nat Rev Microbiol.

[6] Furlan J.P.R., Sellera F.P., Lincopan N., Debone D., Miraglia S.G.E.K., Tavella R.A. (2024). Catastrophic floods and antimicrobial resistance: interconnected threats with wide-ranging impacts. One Health.

[7] Pulingam T., Parumasivam T., Gazzali A.M. (2022). Antimicrobial resistance: prevalence, economic burden, mechanisms of resistance and strategies to overcome. Eur J Pharm Sci.

[8] GBD 2021 Antimicrobial Resistance Collaborators, Vollset S.E., Ikuta K.S. (2024). Global burden of bacterial antimicrobial resistance 1990–2021: a systematic analysis with forecasts to 2050. Lancet.

[9] van Bavel B., Berrang-Ford L., Moon K. (2024). Intersections between climate change and antimicrobial resistance: a systematic scoping review. Lancet Planet Health.

[10] Hashim J.H., Hashim Z. (2016). Climate change, extreme weather events, and human health implications in the Asia Pacific region. Asia Pac J Public Health.

[11] Lai C.-C., Lee K., Xiao Y. (2014). High burden of antimicrobial drug resistance in Asia. J Glob Antimicrob Resist.

[12] Loftus M.J., Stewardson A.J., Naidu R. (2020). Antimicrobial resistance in the Pacific Island countries and territories. BMJ Glob Health.

[13] Yam E.L.Y., Hsu L.Y., Yap E.P.-H. (2019). Antimicrobial resistance in the Asia Pacific region: a meeting report. Antimicrob Resist Infect Control.

[14] Pokharel S., Adhikari B., Johnson T., Cheah P.Y. (2024). Interventions to address antimicrobial resistance: an ethical analysis of key tensions and how they apply in low- income and middle-income countries. BMJ Glob Health.

[15] Mendelson M., Laxminarayan R., Limmathurotsakul D. (2024). Antimicrobial resistance and the great divide: inequity in priorities and agendas between the Global North and the Global South threatens global mitigation of antimicrobial resistance. Lancet Glob Health.

[16] Campbell M., McKenzie J.E., Sowden A. (2020). Synthesis without meta-analysis (SWiM) in systematic reviews: reporting guideline. BMJ.

[17] World Health Organization (2020).

[18] World Health Organization (2020).

[19] World Health Organization (2020).

[20] Boutzoukas A., Doi Y. (2025). The global epidemiology of carbapenem-resistant Acinetobacter baumannii. JAC Antimicrob Resist.

[21] Chen C.H., Wu P.H., Lu M.C., Ho M.W., Hsueh P.R. (2023). Geographic patterns of carbapenem-resistant, multi-drug-resistant and difficult-to-treat Acinetobacter baumannii in the Asia-Pacific region: results from the Antimicrobial Testing Leadership and Surveillance (ATLAS) program, 2020. Int J Antimicrob Agents.

[22] Yang W., Ding L., Han R. (2023). Current status and trends of antimicrobial resistance among clinical isolates in China: a retrospective study of CHINET from 2018 to 2022. One Health Adv.

[23] Argimón S., Masim M.A., Gayeta J.M. (2020). Integrating whole-genome sequencing within the National Antimicrobial Resistance Surveillance Program in the Philippines. Nat Commun.

[24] Foxlee N.D., Townell N., McIver L., Lau C.L. (2019). Antibiotic resistance in Pacific Island countries and territories: a systematic scoping review. Antibiotics (Basel).

[25] World Health Organization (2020).

[26] Broom J., Broom A., Kenny K., Chittem M. (2021). Antimicrobial overuse in India: a symptom of broader societal issues including resource limitations and financial pressures. Glob Public Health.

[27] Nepal G., Bhatta S. (2018). Self-medication with antibiotics in WHO Southeast Asian Region: a systematic review. Cureus.

[28] Lin L., Sun R., Yao T., Zhou X., Harbarth S. (2020). Factors influencing inappropriate use of antibiotics in outpatient and community settings in China: a mixed-methods systematic review. BMJ Glob Health.

[29] McKinn S., Trinh D.H., Drabarek D. (2021). Drivers of antibiotic use in Vietnam: implications for designing community interventions. BMJ Glob Health.

[30] Ferguson J.K., Joseph J., Kangapu S. (2020). Quality microbiological diagnostics and antimicrobial susceptibility testing, an essential component of antimicrobial resistance surveillance and control efforts in Pacific island nations. West Pac Surveill Response J.

[31] Hanna N., Tamhankar A.J., Stålsby Lundborg C. (2023). Antibiotic concentrations and antibiotic resistance in aquatic environments of the WHO Western Pacific and South-East Asia regions: a systematic review and probabilistic environmental hazard assessment. Lancet Planet Health.

[32] Zhen X., Chen J., Sun X., Sun Q., Guo S., Stålsby Lundborg C. (2021). Socioeconomic factors contributing to antibiotic resistance in China: a panel data analysis. Antibiotics (Basel).

[33] Fernández Salgueiro M., Cernuda Martínez J.A., Gan R.K., Arcos González P. (2024). Climate change and antibiotic resistance: a scoping review. Environ Microbiol Rep.

[34] McGough S.F., MacFadden D.R., Hattab M.W., Mølbak K., Santillana M. (2020). Rates of increase of antibiotic resistance and ambient temperature in Europe: a cross-national analysis of 28 countries between 2000 and 2016. Euro Surveill.

[35] MacFadden D.R., McGough S.F., Fisman D., Santillana M., Brownstein J.S. (2018). Antibiotic resistance increases with local temperature. Nat Clim Chang.

[36] Li W., Liu C., Ho H.C. (2023). Association between antibiotic resistance and increasing ambient temperature in China: an ecological study with nationwide panel data. Lancet Reg Health West Pac.

[37] Kaba H.E.J., Kuhlmann E., Scheithauer S. (2020). Thinking outside the box: association of antimicrobial resistance with climate warming in Europe - a 30 country observational study. Int J Hyg Environ Health.

[38] Zhao Y.C., Sun Z.H., Xiao M.X. (2024). Analyzing the correlation between quinolone-resistant Escherichia coli resistance rates and climate factors: a comprehensive analysis across 31 Chinese provinces. Environ Res.

[39] Jian Z., Zeng L., Xu T. (2021). Antibiotic resistance genes in bacteria: occurrence, spread, and control. J Basic Microbiol.

[40] Burnham J.P. (2021). Climate change and antibiotic resistance: a deadly combination. Ther Adv Infect Dis.

[41] Li Z., Sun A., Liu X. (2022). Climate warming increases the proportions of specific antibiotic resistance genes in natural soil ecosystems. J Hazard Mater.

[42] Yu Q., Han Q., Shi S. (2023). Metagenomics reveals the response of antibiotic resistance genes to elevated temperature in the yellow river. Sci Total Environ.

[43] Pietikäinen J., Pettersson M., Bååth E. (2005). Comparison of temperature effects on soil respiration and bacterial and fungal growth rates. FEMS Microbiol Ecol.

[44] Richter K., Haslbeck M., Buchner J. (2010). The heat shock response: life on the verge of death. Mol Cell.

[45] Christaki E., Marcou M., Tofarides A. (2020). Antimicrobial resistance in bacteria: mechanisms, evolution, and persistence. J Mol Evol.

[46] Cohen N.R., Lobritz M.A., Collins J.J. (2013). Microbial persistence and the road to drug resistance. Cell Host Microbe.

[47] Wang Q., Guo S., Hou Z. (2021). Rainfall facilitates the transmission and proliferation of antibiotic resistance genes from ambient air to soil. Sci Total Environ.

[48] Hashimoto M., Hasegawa H., Maeda S. (2019). High temperatures promote cell-to-cell plasmid transformation in Escherichia coli. Biochem Biophys Res Commun.

[49] Anikeeva O., Hansen A., Varghese B. (2024). The impact of increasing temperatures due to climate change on infectious diseases. BMJ.

[50] Cann K., Thomas D.R., Salmon R., Wyn-Jones A., Kay D. (2013). Extreme water-related weather events and waterborne disease. Epidemiol Infect.

[51] Stanke C., Kerac M., Prudhomme C., Medlock J., Murray V. (2013). Health effects of drought: a systematic review of the evidence. PLoS Cur.

[52] Redshaw C.H., Stahl-Timmins W.M., Fleming L.E., Davidson I., Depledge M.H. (2013). Potential changes in disease patterns and pharmaceutical use in response to climate change. J Toxicol Environ Health B Crit Rev.

[53] Serwecińska L. (2020). Antimicrobials and antibiotic-resistant bacteria: a risk to the environment and to public health. Water.

[54] Kolář M., Urbánek K., Látal T. (2001). Antibiotic selective pressure and development of bacterial resistance. Int J Antimicrob Agents.

[55] Holmes A.H., Moore L.S., Sundsfjord A. (2016). Understanding the mechanisms and drivers of antimicrobial resistance. Lancet.

[56] Yarzábal L.A., Salazar L.M.B., Batista-García R.A. (2021). Climate change, melting cryosphere and frozen pathogens: should we worry…?. Environ Sustain (Singap).

[57] Lou Z., Xu H., Xia L., Lin W., Dai Z., Wang X. (2023). Enhanced freeze-thaw cycles facilitate the antibiotic resistance proliferation and dissemination risk under global climate change. Process Saf Environ Prot.

[58] Casadevall A., Kontoyiannis D.P., Robert V. (2019). On the emergence of candida auris: climate change, azoles, swamps, and birds. mBio.

[59] Chakrabarti A., Sood P. (2021). On the emergence, spread and resistance of Candida auris: host, pathogen and environmental tipping points. J Med Microbiol.

[60] The Lancet Regional Health-Western Pacific (2023). Water, climate change, and health in the Western Pacific Region. Lancet Reg Health West Pac.

[61] McMichael C., Dasgupta S., Ayeb-Karlsson S., Kelman I. (2020). A review of estimating population exposure to sea-level rise and the relevance for migration. Environ Res Lett.

[62] Zeng Y., Li W., Zhao M. (2023). The association between ambient temperature and antimicrobial resistance of Klebsiella pneumoniae in China: a difference-in-differences analysis. Front Public Health.

[63] Li W., Liu C., Ho H.C. (2023). Estimating the effect of increasing ambient temperature on antimicrobial resistance in China: a nationwide ecological study with the difference-in-differences approach. Sci Total Environ.

[64] Sun Z.H., Zhao Y.C., Li J.K. (2024). Environmental factors influencing the development and spread of resistance in erythromycin-resistant Streptococcus pneumoniae. Environ Geochem Health.

[65] Zhao Z., Zhang Y., Liu R. (2023). Antibiotic resistance genes in constructed wetlands: driving indicators and risk assessment. J Hazard Mater.

[66] Williams N.L.R., Siboni N., McLellan S.L. (2022). Rainfall leads to elevated levels of antibiotic resistance genes within seawater at an Australian beach. Environ Pollut.

[67] Jang J., Kim M., Baek S. (2021). Hydrometeorological influence on Antibiotic-Resistance genes (ARGs) and bacterial community at a recreational beach in Korea. J Hazard Mater.

[68] Mao G., Ji M., Jiao N. (2023). Monsoon affects the distribution of antibiotic resistome in Tibetan glaciers. Environ Pollut.

[69] Yang Y., Liu G., Ye C., Liu W. (2019). Bacterial community and climate change implication affected the diversity and abundance of antibiotic resistance genes in wetlands on the Qinghai-Tibetan Plateau. J Hazard Mater.

[70] Zheng D., Yin G., Liu M. (2022). Metagenomics highlights the impact of climate and human activities on antibiotic resistance genes in China's estuaries. Environ Pollut.

[71] Yang J.W., Nam J.H., Lee K.J., Yoo J.S. (2024). Effect of temperature on carbapenemase-encoding plasmid transfer in Klebsiella pneumoniae. Microorganisms.

[72] Liu X., Qin P., Wen H., Wang W., Zhao J. (2024). Seasonal meropenem resistance in Acinetobacter baumannii and influence of temperature-driven adaptation. BMC Microbiol.

[73] Sorn S., Sulfikar, Lin M.Y. (2022). Potential impact factors on the enhancement of antibiotic resistance in a Lake environment. J Water Health.

[74] Deng Y., Xu L., Liu S. (2020). What drives changes in the virulence and antibiotic resistance of Vibrio harveyi in the South China Sea?. J Fish Dis.

[75] World Health Organization (2022).

[76] Magnano San Lio R., Favara G., Maugeri A., Barchitta M., Agodi A. (2023). How antimicrobial resistance is linked to climate change: an overview of two intertwined global challenges. Int J Environ Res Public Health.

[77] Yang D., Dyar O.J., Yin J., Ma W., Sun Q., Lundborg C.S. (2024). Antimicrobial resistance in China across human, animal, and environment sectors - a review of policy documents using a governance framework. Lancet Reg Health West Pac.

[78] Robinson T.P., Bu D.P., Carrique-Mas J. (2016). Antibiotic resistance is the quintessential one health issue. Trans R Soc Trop Med Hyg.

[79] Organization WH (2022).

[80] Chua A.Q., Verma M., Hsu L.Y., Legido-Quigley H. (2021). An analysis of national action plans on antimicrobial resistance in Southeast Asia using a governance framework approach. Lancet Reg Health West Pac.

[81] Harring N., Krockow E.M. (2021). The social dilemmas of climate change and antibiotic resistance: an analytic comparison and discussion of policy implications. Hum Soc Sci Commun.

[82] Belachew S.A., Hall L., Erku D.A., Selvey L.A. (2021). No prescription? No problem: drivers of non-prescribed sale of antibiotics among community drug retail outlets in low and middle income countries: a systematic review of qualitative studies. BMC Public Health.

[83] Charani E., Mendelson M., Pallett S.J. (2023). An analysis of existing national action plans for antimicrobial resistance—gaps and opportunities in strategies optimising antibiotic use in human populations. Lancet Glob Health.

[84] Torres N.F., Chibi B., Middleton L.E., Solomon V.P., Mashamba-Thompson T.P. (2019). Evidence of factors influencing self-medication with antibiotics in low and middle-income countries: a systematic scoping review. Public Health.

[85] Kaba H.E.J., Scheithauer S. (2022). Estimating the effect of practicing nursing professionals density on cumulative carbapenem-resistance prevalence in gram-negative invasive Isolates: a 30 European country observational modeling study. Antimicrob Resist Infect Control.

[86] Rameshshanker V., Wyngaarden S., Lau L.L., Dodd W. (2021). Health system resilience to extreme weather events in Asia-Pacific: a scoping review. Clim Dev.

[87] World Health Organization (2016).

[88] World Health Organization (2015).

[89] World Health Organization (2015).

[90] World Health Organization (2020).

[91] Mendelson M., Matsoso M.P. (2015). The World Health Organization Global Action Plan for antimicrobial resistance. S Afr Med J.

[92] Bloom G., Merrett G.B., Wilkinson A., Lin V., Paulin S. (2017). Antimicrobial resistance and universal health coverage. BMJ Glob Health.

[93] Aslam B., Asghar R., Muzammil S. (2024). AMR and sustainable development goals: at a crossroads. Global Health.

