# Supplementary material for: Climate change and antimicrobial resistance in the Western Pacific: a mixed-methods systematic analysis
Source: Lancet Reg Health West Pac. 2025 Dec 16;67:101772. doi: 10.1016/j.lanwpc.2025.101772 (PMC12768945; doi:10.1016/j.lanwpc.2025.101772)
Supplement: Supplementary Material [file mmc1.docx]

**Climate Change and Antimicrobial Resistance in the Western Pacific: A Mixed-Methods Systematic Analysis**

**Supplementary Material**

**[Supplementary Appendix](#_Toc213593420)** [2](#_Toc213593420)

[A. Narrative Review Methodology 2](#_Toc213593421)

[Literature Search strategy 2](#_Toc213593422)

[B. Systematic Review of WPR 3](#_Toc213593423)

[Search Strategy and Selection Criteria 3](#_Toc213593424)

[Inclusion and Exclusion Criteria 4](#_Toc213593425)

[Quality Assessment 4](#_Toc213593426)

[C. Countries, territories and areas in the WHO Western Pacific Region 6](#_Toc213593427)

[Supplementary Figures and Tables 7](#_Toc213593428)

[Figure S1: PRISMA Flow Diagram 7](#_Toc213593429)

[Table S1-S4: Synthesis Without Meta-analysis (SWiM): The Climate-AMR Nexus in the Western Pacific Region 8](#_Toc213593430)

[Table S5-S6: Descriptive of Variables 19](#_Toc213593431)

[Figure S2-S7: Variable correlation matrix 20](#_Toc213593432)

[Table S7: Regression results 23](#_Toc213593433)

[Table S8-S11 Model Diagnosis and Fixed Effects Model 24](#_Toc213593434)

**Supplementary Appendix**

1. **Narrative Review Methodology**

**Literature Search strategy**

We conducted a literature search in PubMed and Google Scholar for articles published up to March 31, 2025. Our search strategy was organized around three core concepts, utilizing a combination of three sets of keywords: (1) “antimicrobial resistance”, “AMR”, “antibiotic resistance”, “antibiotic resistance gene”; (2) "climate change", "global warming," “rising temperature”, “extreme weather”, “heat waves", "flooding"; and (3) “Western Pacific”, “Asia-Pacific," or individual country names. To establish the regional AMR landscape, we combined keywords from categories (1) and (3). To synthesize the global mechanisms linking climate change and AMR, we used keywords from categories (1) and (2). The final selection of references was guided by their direct relevance to these themes. We included observational studies (particularly ecological research), comprehensive reviews, and foundational laboratory studies for mechanistic insights. All literature was published in English, and pre-print articles were excluded.

**Grey literature**

To ensure a comprehensive overview, our search was complemented by a review of grey literature from WHO Institutional Repository for Information Sharing (IRIS)(https://iris.who.int/home). The search strategy was designed to capture all documents linking antimicrobial resistance and climate change within a health context, without geographical or temporal restrictions in the initial search, to maximize sensitivity.

The following search string was developed and executed in the search box, utilizing Boolean operators and wildcard truncation to account for variant terminology:

("antimicrobial resistance" OR "antibiotic resistance" OR AMR OR "antibiotic resistance genes" OR ARG OR "drug resistance") AND ("climate change" OR "global warming" OR temperature OR heatwave OR rainfall OR precipitation OR flood OR drought OR "extreme weather" OR cyclone OR typhoon)

Subsequently, the following filters were applied to the results to refine the search:

language: ("English")

Publication Date: ("2015 - 2025") to capture the modern policy landscape.

All documents in the resulting list were screened by title and abstract for relevance. A document was included for full-text review if it explicitly discussed or presented data on the intersection of antimicrobial resistance and climate or environmental factors. This methodology ensures that our approach is transparent and can be precisely replicated by other researchers.

1. **Systematic Review of WPR**

**Search Strategy and Selection Criteria**

When searching for evidence related to the Western Pacific region, search terms combine climate related and AMR related keywords:

**Pubmed:**

We applied a comprehensive Boolean strategy combining climate-related and antimicrobial resistance–related terms, as well as country and regional identifiers within the WHO Western Pacific Region:

("Climate Change"[Mesh] OR "Climate Change" OR "Climate Changes" OR "Extreme Heat"[Mesh] OR "Extreme Weather" OR "Extreme Heat" OR "Hot Weather" OR "Extreme Heat Wave" OR "Heat Wave" OR "Temperature"[Mesh] OR “Precipitation” OR “Rainfall” OR “Flood” OR “Drought”)  **AND** ("Drug Resistance, Microbial"[Mesh] OR "Microbial Drug Resistance" OR "Antibiotic Resistance, Microbial" OR "Antibiotic Resistance" OR "Antimicrobial Drug Resistance" OR "Antimicrobial Resistance" OR "Antibiotic Resistance Genes") **AND** (“Western Pacific" OR "WHO Western Pacific" OR "Asia-Pacific" OR "Pacific Island" OR "American Samoa" OR “Australia” OR "Brunei Darussalam" OR Cambodia OR “China” OR "Cook Islands" OR “Fiji” OR "French Polynesia" OR “Guam” OR "Hong Kong SAR" OR “Indonesia” OR “Japan” OR Kiribati OR "Lao People's Democratic Republic" OR “Laos” OR "Macao SAR" OR "Macau" OR “Malaysia” OR "Marshall Islands" OR “Micronesia” OR "Federated States of Micronesia" OR “Mongolia” OR “Nauru” OR "New Caledonia" OR "New Zealand" OR “Niue” OR "Northern Mariana Islands" OR “Palau” OR "Papua New Guinea" OR Philippines OR "Pitcairn Islands" OR “Korea” OR “Samoa” OR “Singapore” OR “Solomon Islands” OR “Tokelau” OR “Tonga” OR “Tuvalu” OR “Vanuatu” OR “Viet Nam” OR “Wallis and Futuna”) **AND** ("2000-01-01"[Date - Publication] : "2025-03-31"[Date - Publication]) AND English[lang]

**Google scholar**:

Because of field limitations in Google Scholar, searches were conducted in batches using combined keyword sets for climate and AMR topics and repeated across Western Pacific subregions. The general syntax was:

("antimicrobial resistance" OR "antibiotic resistance" OR AMR OR “antibiotic resistance genes” OR ARG) AND ("climate change" OR "global warming" OR temperature OR heatwave OR rainfall OR precipitation OR flood OR drought OR "extreme weather" OR cyclone OR typhoon) AND ("Western Pacific" OR "WHO Western Pacific" OR "Asia-Pacific" OR "Pacific Islands")

("antimicrobial resistance" OR "antibiotic resistance" OR AMR OR “antibiotic resistance genes” OR ARG) AND ("climate change" OR "global warming" OR temperature OR heatwave OR rainfall OR precipitation OR flood OR drought OR "extreme weather") AND ("American Samoa" OR Australia OR "Brunei Darussalam" OR Cambodia OR China OR "Cook Islands" OR Fiji OR "French Polynesia" OR Guam OR "Hong Kong" OR "Hong Kong SAR")

("antimicrobial resistance" OR "antibiotic resistance" OR AMR OR “antibiotic resistance genes” OR ARG) AND ("climate change" OR "global warming" OR temperature OR heatwave OR rainfall OR precipitation OR flood OR drought OR "extreme weather") AND (Japan OR Kiribati OR "Lao People's Democratic Republic" OR Laos OR "Macao" OR Macau OR Malaysia OR "Marshall Islands" OR Micronesia OR "Federated States of Micronesia" OR Mongolia OR Nauru OR "New Caledonia")

("antimicrobial resistance" OR "antibiotic resistance" OR AMR OR “antibiotic resistance genes” OR ARG) AND ("climate change" OR "global warming" OR temperature OR heatwave OR rainfall OR precipitation OR flood OR drought OR "extreme weather") AND ("New Zealand" OR Niue OR "Northern Mariana Islands" OR Palau OR "Papua New Guinea" OR Philippines OR "Pitcairn Islands" OR "Republic of Korea" OR "South Korea" OR Samoa OR Singapore)

("antimicrobial resistance" OR "antibiotic resistance" OR AMR OR “antibiotic resistance genes” OR ARG) AND ("climate change" OR "global warming" OR temperature OR heatwave OR rainfall OR precipitation OR flood OR drought OR "extreme weather" OR cyclone OR typhoon) AND ("Solomon Islands" OR Tokelau OR Tonga OR Tuvalu OR Vanuatu OR "Viet Nam" OR Vietnam OR "Wallis and Futuna")

**Inclusion and Exclusion Criteria**

**Inclusion criteria:**

- Empirical or modeling studies reporting quantitative or directional associations between climate variables and AMR or ARGs.
- Conducted within countries/territories of the Western Pacific region.
- Published in peer-reviewed journals.

**Exclusion criteria:**

- Studies without primary data (e.g., reviews, commentaries).
- Laboratory or simulation studies not based on real-world data (Purely in vitro experimental studies or laboratory simulation studies that do not utilize environmental or clinical samples, or are not directly relevant to real-world ecological or epidemiological contexts)
- No climate-related exposure variables.
- Studies outside the Western Pacific.
- Qualitative-only or descriptive studies without quantifiable outcomes.

**Quality Assessment**

Two reviewers independently screened titles, abstracts, and full texts. Discrepancies were resolved by discussion. Two reviewers conducted the quality assessment of the included studies independently, and any discrepancies were resolved through mutual agreement. The risk of bias (RoB) was systematically assessed for all 18 included studies, with the appraisal tool or conceptual framework selected based on the study design for non-randomised studies of interventions investigating the causal association between climate exposure and clinical Antimicrobial Resistance (AMR) rates, the conceptual framework of ROBINS-I (Risk of Bias In Non-randomised Studies-of Interventions) was applied. For cross-sectional monitoring studies (e.g., metagenomics surveys), the internal validity criteria from the JBI (Joanna Briggs Institute) Critical Appraisal Checklist for Cross-Sectional Studies or AXIS (Appraisal of Cross-sectional Studies) were applied. For laboratory-based experimental studies, principles from the JBI Checklist for Quasi-Experimental Studies and SYRCLE's RoB Tool were applied. The overall RoB for each study was categorized as Critical, Serious, Moderate, or Low. The final assessment is summarized in Table S1. The certainty of the evidence for the synthesized findings within each One Health category was assessed using the GRADE (Grading of Recommendations Assessment, Development and Evaluation) methodology. The final certainty of evidence was categorized as High, Moderate, Low, or Very Low, as presented in Table S2.

1. **Countries, territories and areas in the WHO Western Pacific Region**^^[[1]](#footnote-0)^^
2. American Samoa (USA)
3. Australia
4. Brunei Darussalam
5. Cambodia
6. China
7. Cook Islands
8. Fiji
9. French Polynesia (France)
10. Guam (USA)
11. Hong Kong SAR (China)
12. Indonesia
13. Japan
14. Kiribati
15. Lao People's Democratic Republic
16. Macao SAR (China)
17. Malaysia
18. Marshall Islands
19. Micronesia, Federated States of
20. Mongolia
21. Nauru
22. New Caledonia (France)
23. New Zealand
24. Niue
25. Northern Mariana Islands (USA)
26. Palau
27. Papua New Guinea
28. Philippines
29. Pitcairn Islands (UK)
30. Republic of Korea
31. Samoa
32. Singapore
33. Solomon Islands
34. Tokelau
35. Tonga
36. Tuvalu
37. Vanuatu
38. Viet Nam
39. Wallis and Futuna (France)

Notes: The Global Antimicrobial Resistance Study (GRAM) in the Western Pacific region has not been included in some areas of the region. Specifically: French Polynesia (France), Hong Kong SAR (China), Macao SAR (China), New Caledonia (France), Pitcairn Islands (UK), Wallis and Futuna (France)

**Supplementary Figures and Tables**

**Figure S1: PRISMA Flow Diagram**

Records identified from Google scholar (n = 66)

Records identified from PubMed (n = 1113)

**Identification**

Duplicates removed (n=17)

Records after duplicates removed (n = 1162)

Records excluded after title/abstract screening (n=1118)

Records screened (n = 1162)

**Screening**

Articles excluded:

Research type mismatch(n=5)

Non-original research(n=3)

Insufficient data for synthesis(n=7)

Non-relevant geographic scope(n=11)

Unavailable or inaccessible full text(n=0)

Full-text articles assessed for eligibility (n=44)

**Included**

Studies included in review (n = 18)

**Figure S1: PRISMA Flow Diagram**

**Table S1-S4: Synthesis Without Meta-analysis (SWiM): The Climate-AMR Nexus in the Western Pacific Region**

**Table S1** **Risk of Bias (RoB) Assessment for Individual Studies (N=18)**

| **Group** | **Title** | **Study Design** | **RoB Tool Used** | **Overall RoB Score** | **Main Limitations and Sources of Bias** |
| --- | --- | --- | --- | --- | --- |
| A: Clinical/Epidemiological Evidence (N=5) | Analyzing the correlation between quinolone-resistant *Escherichia coli* resistance rates and climate factors: A comprehensive analysis across 31 Chinese provinces. | Ecological/Panel Regression | ROBINS-I | Critical | Confounding: High risk of unmeasured/residual confounding (e.g., specific antibiotic consumption, healthcare quality, patient movement).  Ecological Fallacy: Association observed at the regional level may not hold at the individual patient level. |
|  | Environmental factors influencing the development and spread of resistance in erythromycin-resistant *streptococcus pneumoniae* | Ecological/Regression | ROBINS-I | Critical | Confounding: High risk of bias from uncontrolled confounders (e.g., drug prescribing policies).  Exposure Measurement: Climate variables are macro-level surrogates for micro-environmental exposure. |
|  | The association between ambient temperature and antimicrobial resistance of *Klebsiella pneumoniae* in China: a difference-in-differences analysis | Ecological/Difference-in-Differences (DID) | ROBINS-I | Serious | Pre-Intervention Confounding: Reliance on the untestable assumption of parallel trends in the DiD design, which may be violated by differential regional changes.  Ecological Fallacy: Aggregated data limits causal inference for clinical outcomes. |
|  | Estimating the effect of increasing ambient temperature on antimicrobial resistance in China: A nationwide ecological study with the difference-in-differences approach | Ecological/Difference-in-Differences (DID) | ROBINS-I | Serious | Pre-Intervention Confounding: The parallel trends assumption is a major source of bias.  Statistical Assumptions: The complexity of the DiD model may introduce model specification bias. |
|  | Association between antibiotic resistance and increasing ambient temperature in China: an ecological study with nationwide panel data. | Ecological/Panel Data (Log-linear Model) | ROBINS-I | Critical | Confounding: Failure to adjust for all necessary clinical/socioeconomic confounders (e.g., patient severity, hospital type).  Indirectness: Macro-level temperature is an indirect measure of the actual biological exposure. |
| B: Environmental Dissemination Evidence (N=9) | Monsoon affects the distribution of antibiotic resistance in Tibetan glaciers | Environmental Survey/Metagenomics | JBI (Cross-sectional) / AXIS | Low | Sampling Representativeness: Single-time point or limited temporal/spatial sampling in the glacier environment may not fully capture seasonal variability or entire resistome. |
|  | Metagenomics reveals the response of antibiotic resistance genes to elevated temperature in the Yellow River. | Microcosm Experiment/Metagenomics | JBI (Quasi-experimental) | Moderate | Ecological Validity: Controlled microcosm conditions (specific temperature range 23∘C→35∘C, specific duration) simplify the natural river environment, limiting generalizability. |
|  | Antibiotic resistance genes in constructed wetlands: Driving indicators and risk assessment | Environmental Survey/Modeling | JBI (Cross-sectional) / AXIS | Low | Causality: While correlation is strong (R=0.6863), the observational nature of the survey limits the ability to establish a definitive causal chain. |
|  | Metagenomics highlights the impact of climate and human activities on antibiotic resistance genes in China's estuaries | Environmental Survey/Metagenomics | JBI (Cross-sectional) / AXIS | Low | Confounding: Difficulty in completely disentangling the effects of climate factors (wet/dry season) from intense human activities (e.g., sewage input) in estuaries. |
|  | Climate warming increases the proportions of specific antibiotic resistance genes in natural soil ecosystems. | Field Climate Manipulation Experiment/qPCR | JBI (Quasi-experimental) | Low | External Validity: Findings are specific to the tested forest soil and ARG types, and may not generalize to other soil types or ecosystems. |
|  | Hydrometeorological Influence on Antibiotic-Resistance Genes (ARGs) and Bacterial Community at a Recreational Beach in Korea | Environmental Survey/Modeling | JBI (Cross-sectional) / AXIS | Low | Measurement Frequency: ARG levels were measured less frequently than key hydrometeorological factors, potentially masking short-term fluctuation impacts. |
|  | Bacterial community and climate change implication affected the diversity and abundance of antibiotic resistance genes in wetlands on the Qinghai Tibetan Plateau | Environmental Survey/Modeling | JBI (Cross-sectional) / AXIS | Low | Geographic Scope: Large geographic area (Qinghai-Tibetan Plateau) with high variation in environmental factors, making generalized conclusions prone to heterogeneity bias. |
|  | Rainfall leads to elevated levels of antibiotic resistance genes within seawater at an Australian beach | Environmental Survey/Modeling | JBI (Cross-sectional) / AXIS | Low | Indirectness: The link to public health risk relies on the assumed connection between rainfall, sewage overflow, elevated ARGs, and human exposure. |
|  | Rainfall facilitates the transmission and proliferation of antibiotic resistance genes from ambient air to soil | Field Survey | JBI (Quasi-experimental) | Low | Transferability: The findings regarding air-to-soil transfer are specific to the heavy metal-contaminated soil used in the study, limiting generalizability to clean environments. |
| C: Mechanistic/Biological Plausibility (N=4) | Effect of Temperature on Carbapenemase-Encoding Plasmid Transfer in *Klebsiella pneumoniae* | In Vitro Laboratory Experiment | SYRCLE's RoB / JBI (Quasi-experimental) | Low | Indirectness (Ecological Validity): Highly controlled, simplified in vitro system (pure culture, specific media) does not replicate the competition, phage, and environmental pressures of a natural or host environment. |
|  | Seasonal meropenem resistance in *Acinetobacter baumanni*i and influence of temperature-driven adaptation | Ecological Survey + Lab Experiment | ROBINS-I + SYRCLE's RoB | Moderate | Combined RoB: The ecological part shares Critical RoB (confounding/fallacy), while the lab part has Low RoB but High Indirectness (cold stress (4∘C) is extreme and not fully representative of seasonal change). |
|  | Potential impact factors on the enhancement of antibiotic resistance in a lake environment | Microcosm Experiment (Lake Simulation) | JBI (Quasi-experimental) | Low | Indirectness: The use of relatively high norfloxacin concentrations (ng/L) for induction, while low in absolute terms, may be higher than average ambient levels, potentially exaggerating the effect size. |
|  | What drives changes in the virulence and antibiotic resistance of *Vibrio harveyi* in the South China Sea? | Field Survey/Lab | JBI (Cross-sectional) | Moderate | Confounding: The correlation between warmer waters and increased resistance in V. harveyi is highly confounded by the differential level of aquaculture/antibiotic use between the Hainan and Guangdong sites. |

**Table S2** **GRADE Assessment of Categorized Evidence**

| **One Health Evidence Category**  **(Representative PICO)** | **Initial Design Certainty** | **Risk of Bias**  **(RoB) / Study Limitations** | **Inconsistency** | **Indirectness** | **Imprecision** | **Publication Bias** | **Upgrading Factors** | **Final Certainty** |
| --- | --- | --- | --- | --- | --- | --- | --- | --- |
| A: Clinical/Epidemiological Evidence(N=5) | Low (Ecological/Time-Series/Observational) | Serious: High risk of unmeasured confounding (e.g., actual antibiotic consumption, healthcare quality, population density, mobility). Ecological fallacy risk is inherent. | Not Serious: Most studies consistently show a positive association between rising temperature and clinical AMR rates (e.g., *K. pneumoniae*, *E. coli*). | Serious: Exposure is macro-level climate (ambient temp) not an intervention. Outcome is aggregated resistance rate (surrogate) not patient-level morbidity/mortality. | Not Serious: Nationwide panel data with large sample sizes (across provinces/years) provides sufficient power. | Not Serious/Unlikely: Signal is consistent across multiple studies. | 1. Large Magnitude of Effect: DiD models often show statistically significant, substantial positive effect estimates. | Low |
| B: Environmental Dissemination Evidence(N=9) | Low (Environmental Monitoring/Metagenomics) | Not Serious: Studies typically use validated molecular/sequencing methods, minimizing internal bias; confounding is addressed by statistical modeling (e.g., variation partitioning). | Not Serious: High consistency. Multiple studies across diverse environments (glacier, river, soil, wetland, estuary) consistently identify temperature/hydrology as the primary drivers of ARG changes. | Serious: Outcome (ARGs abundance/diversity) is a distal surrogate for the final public health outcome (clinical infection). The link requires multiple steps (transmission, exposure dose, infection). | Not Serious: Metagenomics data provides deep coverage, leading to precise estimates of resistome profiles. | Not Assessable | 1. High Consistency / Plausibility: The effect is consistently demonstrated in multiple, heterogeneous settings.   2. Large Magnitude of Effect: Climate factors often explain the largest proportion of total variance in ARG profiles. | Moderate |
| C: Mechanistic/Biological Plausibility(N=4) | High (Controlled *In Vitro*/Experimental) | Not Serious: Highly controlled lab environments ensure minimal confounding and high internal validity. | Not Serious: Findings are consistent: higher temperature promotes HGT (plasmid transfer) and alters bacterial adaptation/virulence. | Serious: High Indirectness (Lack of ecological validity). Experimental conditions (pure culture, artificial media, simplified environment) are highly dissimilar to the complex *in vivo* or natural ecosystems. | Not Serious: Controlled experiments are designed for high precision. | Not Assessable | 1. Dose-Response Gradient: HGT frequency clearly increases with rising temperature.   2. Biological Plausibility: Provides direct, strong biological evidence for mechanisms underlying A and B categories. | Moderate |

**Table S3** **Characteristics of Included Studies (N=18)**

| **Group** | **Paper ID** | **Title** | **Author**  **(Year)** | **Study Design** | **Location** | **Sample Type** | **Exposure(Climate Factor)** | **AMR Indicator** | **Core Finding** |
| --- | --- | --- | --- | --- | --- | --- | --- | --- | --- |
| A: Clinical/Epidemiological Evidence (N=5) | 1 | Analyzing the correlation between quinolone-resistant *Escherichia coli* resistance rates and climate factors: A comprehensive analysis across 31 Chinese provinces. | Zhao Y, et al.(2024) | Ecological/Panel Regression | 31 provinces and cities within China mainland | Data on E. coli from the China Antimicrobial Resistance Surveillance System (CARSS) | Annual/Seasonal Temperature and Rainfall | Quinolone-resistant *E.coli* (QnR eco) resistance rates and detection rates | The study found higher resistance rates in warmer, monsoon climates and areas with more public health facilities, but lower rates in cooler, mountainous, or continental climates with more rainfall; The predictive model for resistance rates, integrating climate and healthcare factors, explained 64.1% of the variance (adjusted R-square = 0.641). |
|  | 2 | Environmental factors influencing the development and spread of resistance in erythromycin-resistant *streptococcus pneumoniae* | Sun Z, et al.(2024) | Ecological/Regression | China (Nationwide) | Data on *Streptococcus pneumoniae* from the China Antimicrobial Resistance Surveillance System. | Geographic and Climatic Factors (Temperature) | Drug resistance rate and isolate rate of erythromycin-resistant *SP* | Multivariate regression analysis indicated positive correlations between the drug resistance rate and temperature, Subtropical climate, Gross Domestic Product (GDP), Hu Huanyong line, and the highest temperature in the past period (Tm). |
|  | 3 | The association between ambient temperature and antimicrobial resistance of *Klebsiella pneumoniae* in China: a difference-in-differences analysis | Zeng, Y.C., et al.(2023) | Ecological/Difference-in-Differences (DiD) | 31 provinces in China | Provincial antimicrobial resistance surveillance data from hospitals (1,371 hospitals including tertiary and secondary hospitals) | Ambient Temperature | Detection rate of third-generation cephalosporin-resistant *K. pneumoniae* (3GCRKP) and carbapenem-resistant *K. pneumoniae* (CRKP) | Every 1°C increase in annual average temperature was associated with a 4.7% increase in 3GCRKP (RR: 1.047, 95% CI: 1.031–1.082) and a 10.7% increase in CRKP (RR: 1.107, 95% CI: 1.011–1.211). Socioeconomic status moderated these associations. |
|  | 4 | Estimating the effect of increasing ambient temperature on antimicrobial resistance in China: A nationwide ecological study with the difference-in-differences approach | Li W, et al.(2023) | Ecological/Difference-in-Differences (DID) | 31 provinces/regions in mainland China | Bacterial isolates collected from over 1000 hospitals across all of the 31 provinces/regions (from the China Antimicrobial Resistance Surveillance System - CARSS) | Ambient Temperature (Increasing trend) | Prevalence of *E. coli* resistance to third-generation cephalosporins (ceftriaxone and cefotaxime, 3GCREC), carbapenems (CREC) and quinolones (QREC) | Every 1 °C increase in average ambient temperature was associated with an increase of 2.71% (95% CI: 1.20–4.24) in 3GCREC, 32.92% (95% CI: 15.62–52.81) in CREC, and 1.81% (95% CI: 0.47–3.16) in QREC. The association was more profound in regions with lower temperature and lower socioeconomic status. |
|  | 5 | Association between antibiotic resistance and increasing ambient temperature in China: an ecological study with nationwide panel data. | Li W, et al.(2023) | Ecological/Panel Data (Log-linear Model) | China (28 provinces/autonomous regions) | Clinical isolates from hospitals participating in the China Antimicrobial Surveillance Network (CHINET) | Ambient Temperature and its change over time | Prevalence of antibiotic resistance (detection rates of CRAB, CRKP, CRPA) | A 1 °C increase in average ambient temperature was associated with 1.14-fold increase (95%-CI [1.07–1.23]) in CRKP prevalence and 1.06-fold increase （95%-CI [1.03–1.08]） in CRPA prevalence. Year-by-year changes in ambient temperature had an accumulative effect, with the four-year sum showing the greatest effect. |
| B: Environmental Dissemination Evidence (N=9) | 6 | Monsoon affects the distribution of antibiotic resistance in Tibetan glaciers | Mao G, et al.(2023) | Environmental Survey/Metagenomics | 21 Tibetan glaciers on the Tibetan Plateau, China | 85 samples of surface snow, ice, and cryoconite | Monsoon and Westerly Circulation | Diversity, abundance, and distribution of ARGs; Co-occurrence of ARGs with mobile genetic elements (MGEs) | The relative abundance of ARGs in snow was significantly higher in monsoon-dominated glaciers than in westerly-dominated glaciers. 90 ARGs were linked to MGEs, and monsoon-dominated glaciers presented a higher risk of releasing MGE-associated multidrug-resistant bacteria. |
|  | 7 | Metagenomics reveals the response of antibiotic resistance genes to elevated temperature in the Yellow River. | Yu Q, et al.(2023) | Microcosm Experiment/Metagenomics | Lanzhou section of the Yellow River, China | Water samples from the Yellow River | Gradually elevated water temperature | ARG diversity, abundance (TPM), types, high-risk ARGs, co-occurrence with opportunistic pathogens | Gradually increased temperature remarkably reduced ARG diversity but increased ARG abundance. For each 1 °C increase, total ARG abundance increased by 2133 TPM. The abundance of five high-risk ARGs (*tetM, mecA, bacA, vatE, tetW*) significantly increased with temperature and co-occurred with opportunistic pathogens. |
|  | 8 | Antibiotic resistance genes in constructed wetlands: Driving indicators and risk assessment | Zhao Z, et al.(2023) | Environmental Survey/Modeling | Constructed wetlands in Tianjin Binhai Industrial Park, China | Water samples collected from constructed wetlands (CW01–CW10) | Seasonal Variation (Temperature) | Absolute abundance of ARGs and MGEs (copies/mL); risk index (RI) of ARGs | Temperature was the main driving factor of ARGs (0.6863) and MGEs (0.5929). ARG abundance was highest in summer. Seasonal variation significantly influenced ARG and MGE abundance and co-occurrence patterns. |
|  | 9 | Metagenomics highlights the impact of climate and human activities on antibiotic resistance genes in China's estuaries | Zheng D, et at.(2022) | Environmental Survey/Metagenomics | 16 China’s estuaries across 6 climate zones | Estuarine sediment samples | Climate Zones, Dry vs. Wet Seasons (Precipitation) | Normalized abundance, richness, and diversity of ARGs (ppm); absolute abundance of ARGs (copies/g); core resistome | ARG abundance, richness, and diversity were higher in dry season than wet season; Climate variables explained 44% of ARG variance, with temperature as the most important climate driver (36% of variance); Temperature was negatively associated with ARG abundance, richness, and diversity. |
|  | 10 | Climate warming increases the proportions of specific antibiotic resistance genes in natural soil ecosystems. | Li Z, et al.(2022) | Field Climate Manipulation Experiment/qPCR | Sanming Forest Ecosystem and Global Change National Observation and Research Station, Fujian Province, China | Forest soil samples (0-15 cm surface soil) | Climate Warming (Warming Shelters) | Proportion (relative abundance) of ARGs (ARGs / 16S rRNA gene); number of ARGs | Climate warming increased the proportions of specific ARGs in forest soils, with these effects being highly dependent on the season. |
|  | 11 | Hydrometeorological Influence on Antibiotic-Resistance Genes (ARGs) and Bacterial Community at a Recreational Beach in Korea | Jang J, et al.(2021) | Environmental Survey/Modeling | Gwangalli Beach, Busan, South Korea | Seawater samples | Hydrometeorological Influence (Rainfall, Tides) | Relative abundance of ARGs (copies/mL); number of ARGs; diversity of ARGs (Shannon's index) | The study found a substantial influence of rainfall and tidal levels on the relative abundance of total ARGs and bacterial operational taxonomic units (OTUs), which showed 1.9×10^3^ and 1.1×10^1^ fold increases, respectively. The elevated levels of ARGs were maintained for up to 32 hours after rainfall. |
|  | 12 | Bacterial community and climate change implication affected the diversity and abundance of antibiotic resistance genes in wetlands on the Qinghai Tibetan Plateau | Yang Y, et al.(2019) | Environmental Survey/Modeling | Qinghai-Tibetan Plateau Wetlands, China | Soil samples from riverine, lacustrine, palustrine wetlands | Geographic Distribution, Climate Change Implication (Soil Moisture) | ARG abundance; diversity; MGEs | Climate change and anthropogenic factors indirectly influence the ARG profile and distribution via the bacterial community. The warming climate trend in the Qinghai-Tibetan will very likely lead to increased soil temperature and moisture which will alter the microbial community composition and increase the background levels of ARGs in the wetlands. |
|  | 13 | Rainfall leads to elevated levels of antibiotic resistance genes within seawater at an Australian beach | Williams, N.R.L, et al. (2022) | Environmental Survey/Modeling | Terrigal Beach, Australia | Seawater; stormwater drains; lagoon water | Rainfall Events, Sewage Contamination | ARG abundance; sewage markers (Lachno3, intI1) | Rainfall led to elevated levels of ARGs and enteric bacteria in seawater, establishing a clear link between precipitation events and contamination of public waters. |
|  | 14 | Rainfall facilitates the transmission and proliferation of antibiotic resistance genes from ambient air to soil | Wang Q, et al. (2021) | Field Survey | Handan, China | PM2.5; rainwater; soil (park and heavy metal-contaminated) | Rainfall, Wind, PM Concentrations | ARG abundance; MGEs; MIC of antibiotics | Rainfall acts as a mobile reservoir, facilitating the transmission and proliferation of ARGs from ambient air to soil environments. |
| C: Mechanistic/Biological Plausibility (N=4) | 15 | Effect of Temperature on Carbapenemase-Encoding Plasmid Transfer in *Klebsiella pneumoniae* | Yang, J. W, et al.(2024) | In Vitro Laboratory Experiment | South Korea (sentinel hospitals in eight regions across the Korean Peninsula) | Clinical isolates of Klebsiella pneumoniae from blood and urine samples | Local temperature (monthly average temperature) | Antibiotic resistance rates; conjugation frequency; plasmid stability | The resistance rate of *K. pneumoniae* in Korea differed significantly across temperature ranges based on local temperature. The study also revealed clear differences in the transfer of plasmids carrying the carbapenemase gene depending on temperature. |
|  | 16 | Seasonal meropenem resistance in *Acinetobacter baumanni*i and influence of temperature-driven adaptation | Liu X, et al.(2024) | Ecological Survey +Laboratory Experiment | Hebei Province, China (data from 45 hospitals). | Clinical isolates of *Acinetobacter baumannii*. | Temperature (local air temperature; low-temperature stress at 4°C in experiments). | Meropenem resistance rate; expression of carbapenem resistance-related genes. | Meropenem resistance rates peaked in winter (≈65 %) and dropped in summer (≈45 %), negatively correlating with temperature (r = -0.69); resistant strains survived better at 4 °C, up-regulated carbapenem-resistance genes (adeJ, oxa-51, oxa-23), and outcompeted sensitive strains under cold stress, explaining the seasonal pattern. |
|  | 17 | Potential impact factors on the enhancement of antibiotic resistance in a lake environment | S Sorn, et al.(2022) | Microcosm Experiment (Lake Simulation) | Kahokugata Lake, Ishikawa Prefecture, Japan | Escherichia coli isolates from lake water samples | Variable Temperatures | Minimum inhibitory concentration (MIC) of norfloxacin; relative MIC changes (fold) | The induction of antibiotic resistance was more likely to occur at 37 °C. Resistance also occurred at 25 °C with longer exposure time (5 days). Higher temperature enhanced resistance in more isolates and increased MIC fold changes. |
|  | 18 | What drives changes in the virulence and antibiotic resistance of *Vibrio harveyi* in the South China Sea? | Deng et al. (2020) | Field Survey/Lab | South China Sea | Vibrio harveyi strains from diseased marine fish | Warmer temperature; antibiotic pollutants | Antibiotic resistance; virulence genes; multidrug resistance | There were more virulence genes and greater drug resistance in Hainan than in Guangdong, suggesting that warmer temperature and antibiotics pollutants probably enhance antibiotic resistance and bacterial infection. |

**Table S4** **Synthesis of Findings on the Climate-AMR Nexus in the Western Pacific Region**

| **One Health Evidence Category**  **(N)** | **Paper ID*** | **Climate Factor**  **(Exposure)** | **AMR Outcome**  **(Indicator)** | **Direction & Association** |
| --- | --- | --- | --- | --- |
| A: Clinical/Epidemiological Evidence (N=5) | 1, 2, 3, 4, 5 | Elevated Ambient Temperature (Mean Monthly/Annual Temp) | Clinical Resistance Rate (e.g., CRAB, CRKP, QnR Eco, ERSP) | **Positive Association**: Rising temperature is consistently and significantly associated with increased clinical resistance rates. |
|  | 1, 2 | Rainfall/Monsoon | Clinical Resistance Rate (QnR Eco, ERSP) | **Complex/Mixed Association**: Positive, negative, and non-linear associations observed depending on region. |
| B: Environmental Dissemination Evidence (N=9) | 6, 7, 8, 9, 10, 11, 12, 13, 14 | Elevated Environmental Temperature (Water/Soil Temp) | Environmental Antibiotic Resistance Gene (ARG) Abundance/Diversity | **Predominantly Positive Association**: Elevated temperature generally leads to increased ARG abundance and/or enhanced dissemination mechanisms. |
|  | 6, 9, 11, 13, 14 | Rainfall/Hydrological Events | Environmental ARG Abundance/Dissemination | **Predominantly Positive Association**: Rainfall acts as a key driver for ARG dispersal from sources into the environment. |
| C: Mechanistic/Biological Plausibility (N=4) | 15, 16, 17, 18 | Elevated Temperature (≥25∘C) | Plasmid Transfer/Resistance Induction/Virulence | **Positive Association**: Promotes Horizontal Gene Transfer (HGT) and enhances bacterial virulence/adaptation. |
|  | 16 | Lower Temperature (≤4∘C) (Cold Stress) | Bacterial Fitness/Survival Advantage | **Positive Association** (Selective): Cold stress selectively benefits resistant strains by enhancing their survival. |

*: Paper ID as shown in the Table S3 Characteristics of Included Studies (N=18).

**Table S5-S6: Descriptive of Variables**

**Table S5 Descriptive of Data Sources**

| Variable | Measurement Unit | Data Sources |
| --- | --- | --- |
| Mean Ambient Temperature (TMP) | ℃ | Climatic Research Unit Time series |
| Precipitation (PREC) | mm | Climatic Research Unit Time series |
| Population-Weighted PM2.5 (PM2.5) | (ug/m3) | Atmospheric Composition Analysis Group |
| Population Density (PD) | people·/km | World Development Indicators in DataBank |
| Hospital Beds (HD) | Per 1,000 population | World Development Indicators in DataBank |
| Health Expenditure (HEG) | % of GDP | World Development Indicators in DataBank |
| Urban Population (UP) | % of total polulation | World Development Indicators in DataBank |
| Corruption Perceptions Index (CPI) | Index·(1-100) | Transparency International |
| Antimicrobial Consumption (AMC) | DDD/1000/day | World Development Indicators in DataBank |
| Water, Sanitation, and Hygiene (WASH) | % total Population | ResistanceMap (IQVIA), Global antimicrobial resistance and use surveillance system (GLASS), European Surveillance of Antimicrobial Consumption Network (ESAC-Net), Global Research on Antimicrobial Resistance (GRAM) project |

**Table S6 Descriptive Statistics for Variables**

| **Variable** | **Mean (Sd)** | **Median (IQR)** | **Skew** |
| --- | --- | --- | --- |
| Mean Ambient Temperature (TMP) | 21.36 (7.10) | 25.00 (12.80-26.80) | -0.77 |
| Precipitation (PREC) | 1560.26 (972.67) | 1533.60 (665.25-2019.45) | 0.74 |
| Population-Weighted PM2.5 (PM2.5) | 19.16 (10.22) | 18.70 (12.45-23.80) | 1.15 |
| Population Density (PD) | 689.80 (1852.70) | 129.97 (67.75-344.93) | 3.20 |
| Hospital Beds (HD) | 3.49 (3.76) | 2.13 (1.06-3.84) | 1.83 |
| Health Expenditure (HEG) | 5.19 (2.69) | 4.17 (3.06-7.27) | 0.77 |
| Urban Population (UP) | 62.43 (25.37) | 70.50 (41.19-85.12) | -0.23 |
| Corruption Perceptions Index (CPI) | 50.70 (24.96) | 41.00 (29.25-74.50) | 0.49 |
| Antimicrobial Consumption (AMC) | 15.61 (7.80) | 15.90 (8.05-22.00) | 0.29 |
| Water, Sanitation, and Hygiene (WASH) | 63.94 (14.52) | 53.64 (53.64-78.07) | 1.03 |


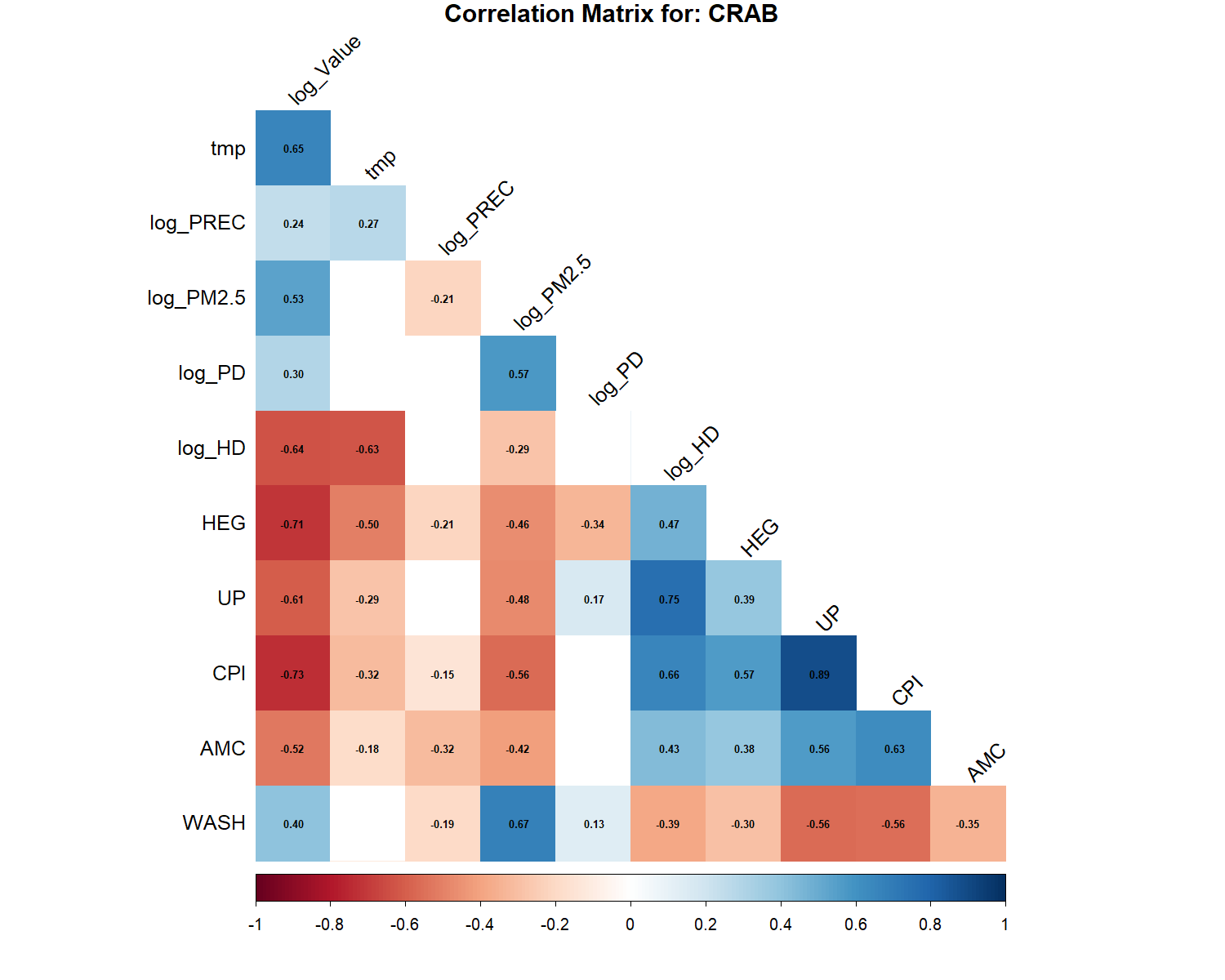
**Figure S2-S7: Variable correlation matrix**

**Figure S2 Correlation Matrix of Key Variables (CRAB)**


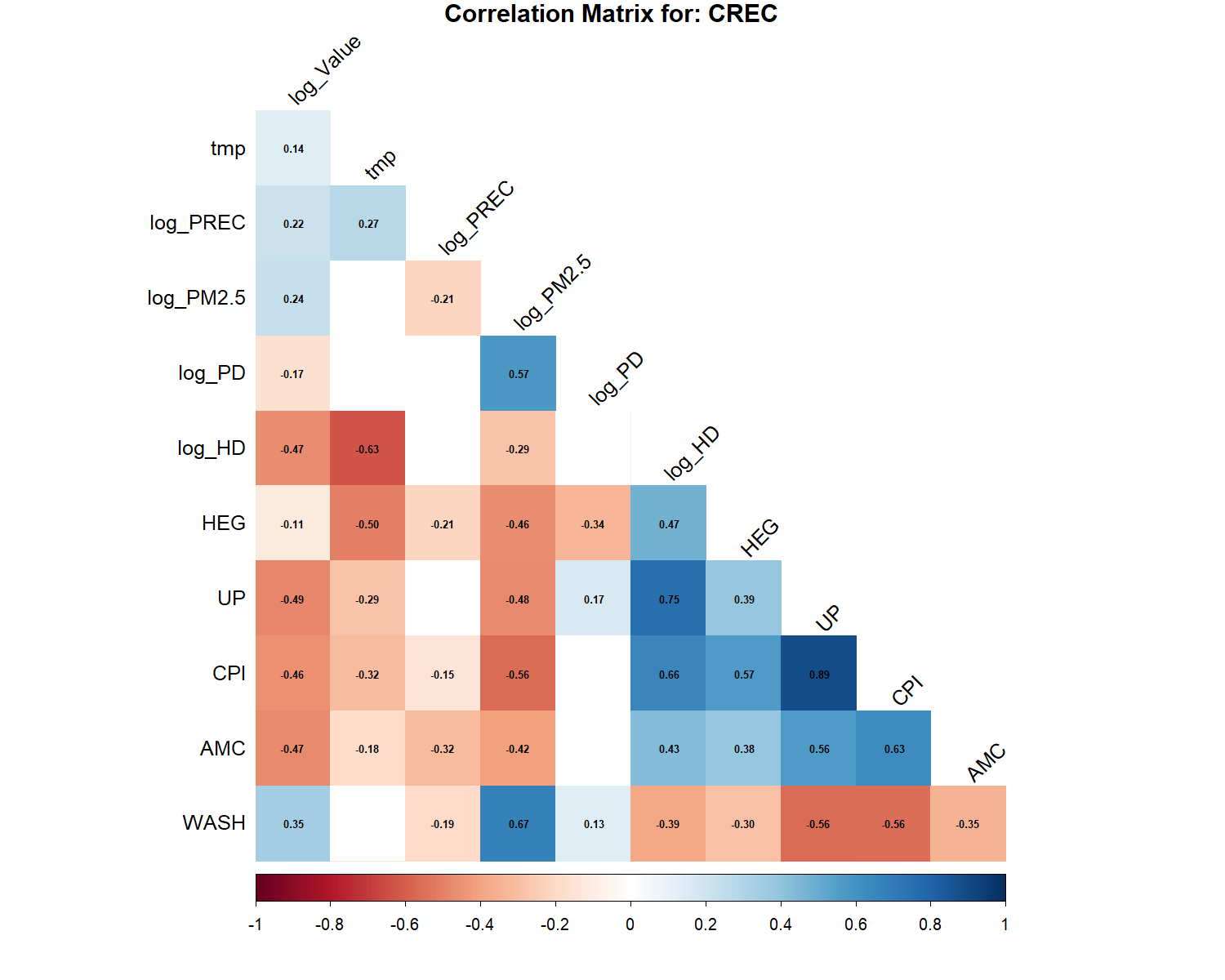


**Figure S3 Correlation Matrix of Key Variables (CREC)**


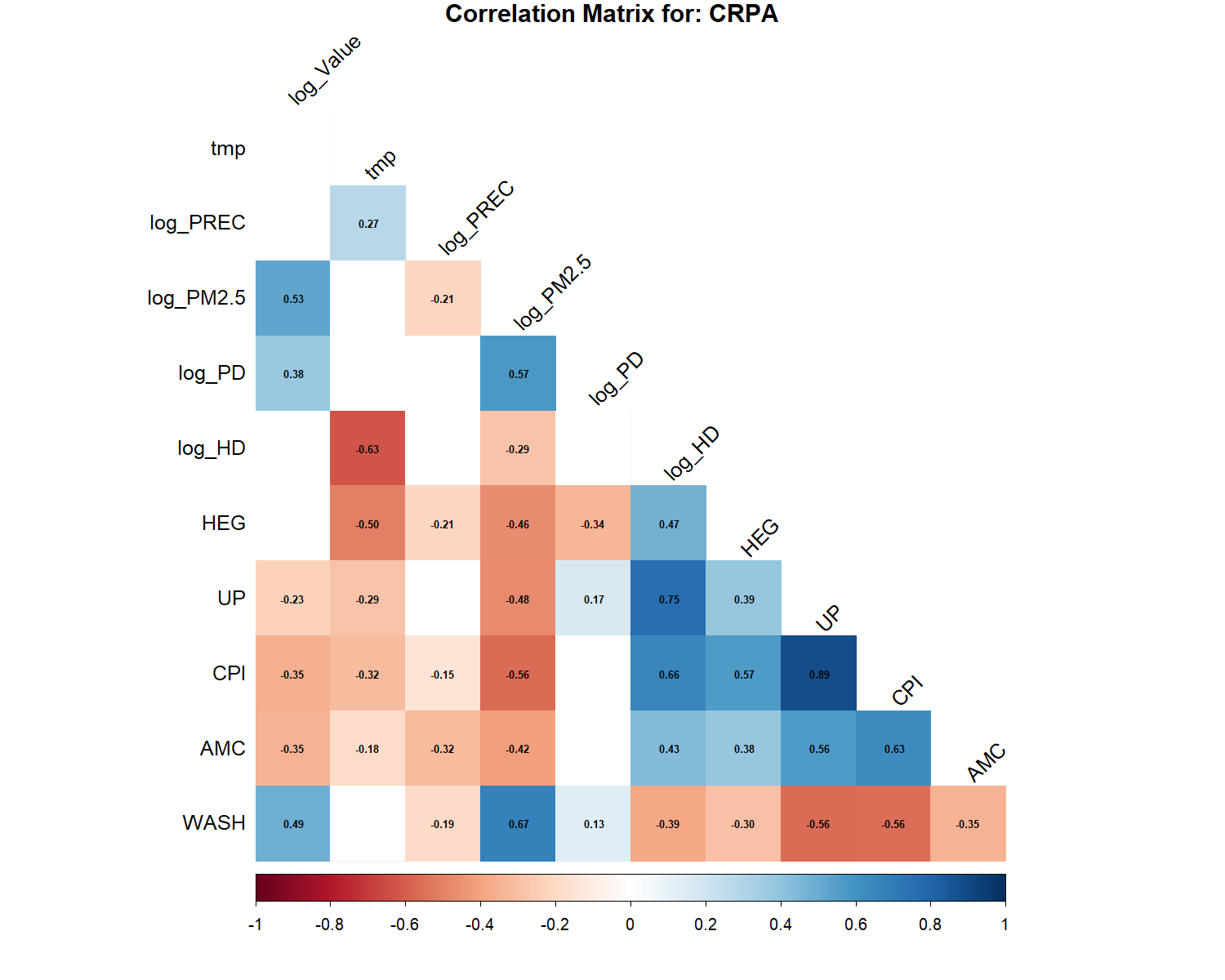

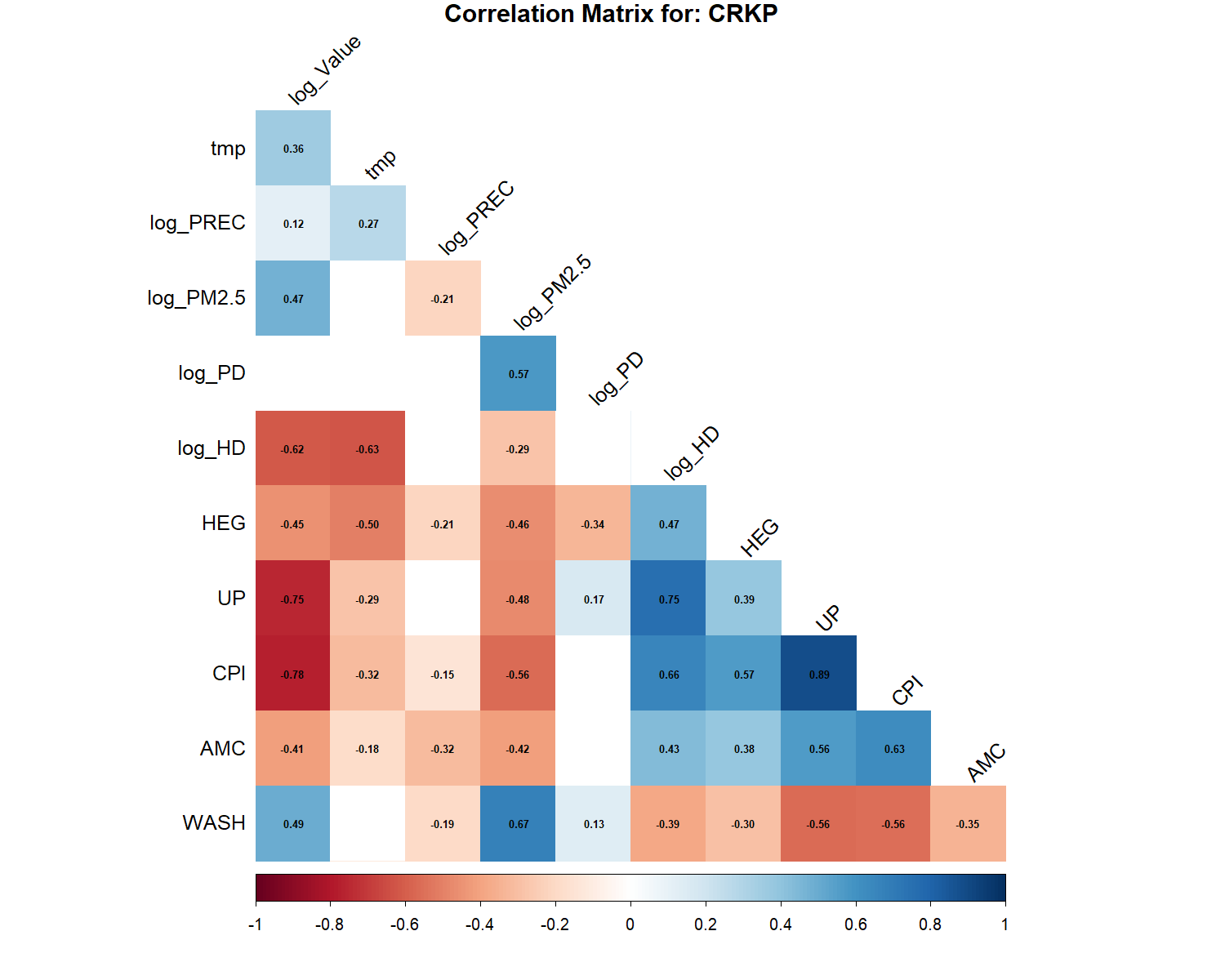
**Figure S4 Correlation Matrix of Key Variables (CRKP)**

**Figure S5 Correlation Matrix of Key Variables (CRPA)**


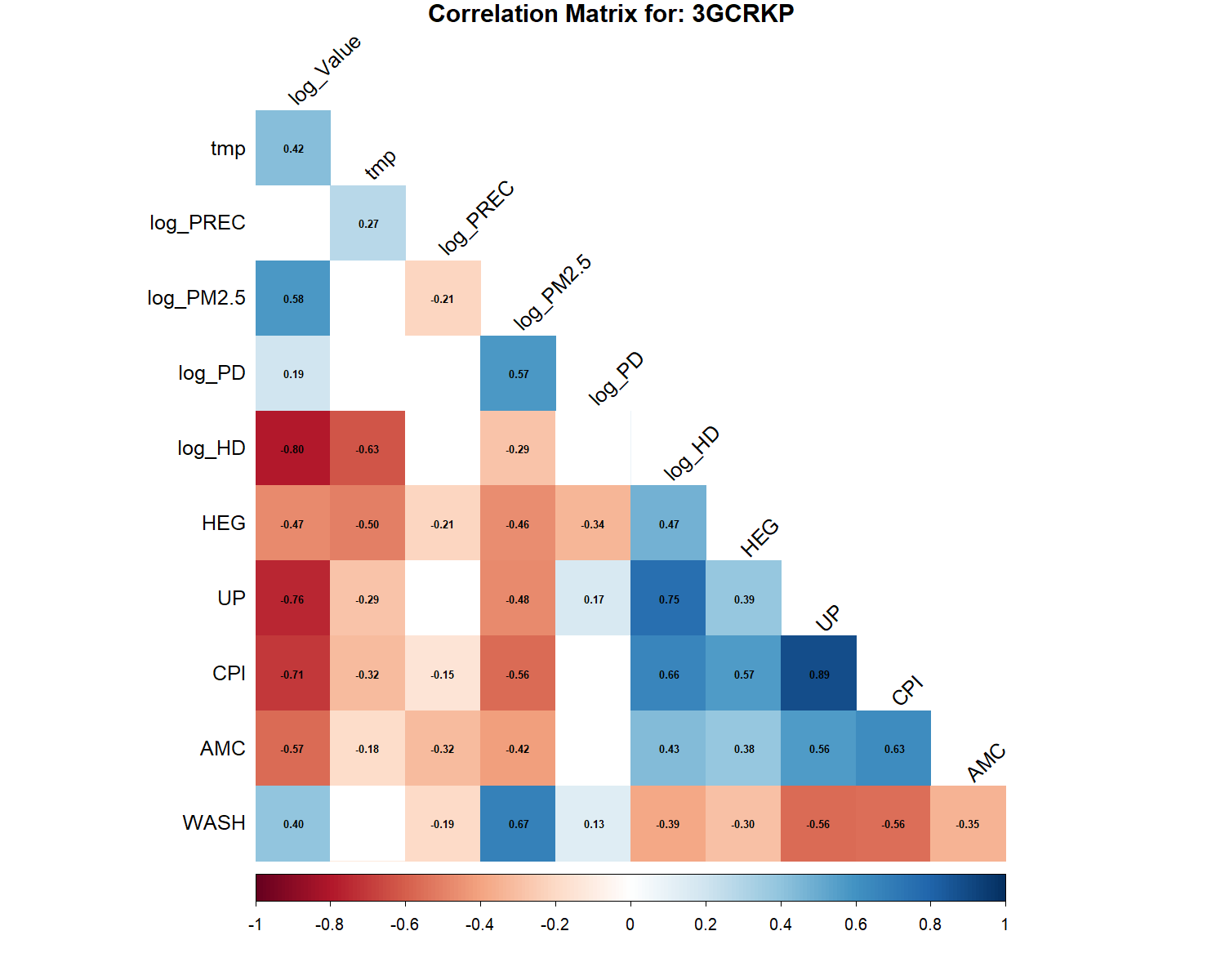

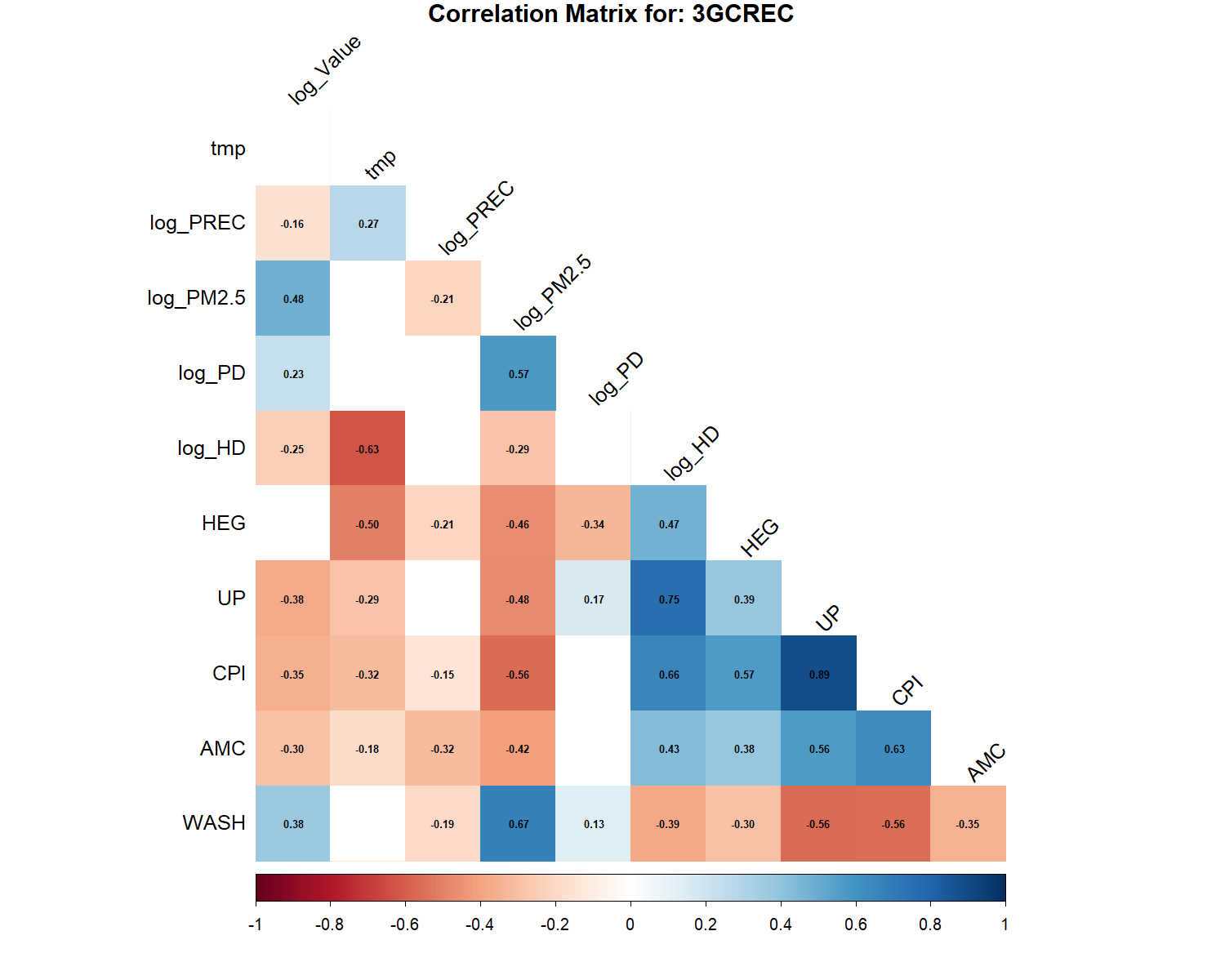
**Figure S6 Correlation Matrix of Key Variables (3GCREC)**

**Figure S7 Correlation Matrix of Key Variables (3GCRK**

|  | **CREC** | |  | **CRKP** | |  | **3GCREC** | |
| --- | --- | --- | --- | --- | --- | --- | --- | --- |
|  | *β [95% CI]* | *P-value* |  | *β [95% CI]* | *P-value* |  | *β [95% CI]* | *P-value* |
| Tmp | 0.058 [-0.101, 0.217] | 0.472 |  | 0.153 [0.046, 0.261] | 0.005 |  | 0.299 [0.130, 0.468] | 0.001 |
| log_PREC | 0.280 [0.160, 0.400] | <0.001 |  | 0.106 [0.026, 0.185] | 0.009 |  | -0.088 [-0.210, 0.034] | 0.156 |
| log_PM2.5 | 0.399 [0.118, 0.679] | 0.006 |  | 0.074 [-0.115, 0.263] | 0.443 |  | 0.380 [0.066, 0.694] | 0.018 |
| log_PD | -0.317 [-0.505, -0.129] | 0.001 |  | 0.084 [-0.044, 0.212] | 0.196 |  | 0.172 [-0.001, 0.345] | 0.052 |
| log_HD | -0.345 [-0.518, -0.172] | <0.001 |  | 0.005 [-0.129, 0.139] | 0.942 |  | 0.092 [-0.076, 0.259] | 0.283 |
| HEG | 0.304 [0.178, 0.431] | <0.001 |  | 0.059 [-0.019, 0.137] | 0.137 |  | 0.483 [0.334, 0.632] | <0.001 |
| UP | 0.160 [-0.121, 0.440] | 0.263 |  | -0.361 [-0.602, -0.121] | 0.003 |  | -0.222 [-0.485, 0.042] | 0.099 |
| CPI | -0.091 [-0.341, 0.159] | 0.474 |  | -0.444 [-0.626, -0.262] | <0.001 |  | -0.048 [-0.311, 0.215] | 0.719 |
| AMC | -0.167 [-0.292, -0.042] | 0.009 |  | 0.172 [0.074, 0.270] | 0.001 |  | -0.149 [-0.265, -0.034] | 0.012 |
| WASH | 0.120 [-0.029, 0.270] | 0.115 |  | 0.094 [-0.021, 0.210] | 0.109 |  | 0.090 [-0.040, 0.219] | 0.175 |
| R2 | 0.43 |  |  | 0.67 |  |  | 0.39 |  |
| adj_R2 | 0.41 |  |  | 0.66 |  |  | 0.37 |  |
| N | 299 |  |  | 299 |  |  | 299 |  |

**Table S7: Regression results**

**Table S7 Regression results of CREC, CRKP and 3GCREC**

**Table S8-S11 Model Diagnosis and Fixed Effects Model**

Considering the presence of unobserved influencing factors, we conducted panel data analyses to account for potential spatial-temporal heterogeneity. First, we performed several diagnostic tests to determine the most appropriate model specification. Both the F-test and Hausman test indicated that a Fixed Effects (FE) model is the most suitable choice over a standard Pooled OLS or Random Effects (RE) model. Based on these results, we proceeded with individual fixed effects modeling and two-way fixed effects modeling.

**Table S8 Comparison of the within and the pooling model.**

| **F test** | **CRAB** | | | |  | **CREC** | | | |  | **CRKP** | | | |
| --- | --- | --- | --- | --- | --- | --- | --- | --- | --- | --- | --- | --- | --- | --- |
|  | *F* | *df1* | *df2* | *p* |  | *F* | *df1* | *df2* | *p* |  | *F* | *df1* | *df2* | *p* |
| Individual effects | 41.85 | 12 | 276 | <0.001 |  | 14.98 | 12 | 276 | <0.001 |  | 14.25 | 12 | 276 | <0.001 |
| Time effects | 0.41 | 22 | 266 | 0.992 |  | 0.41 | 22 | 266 | 0.992 |  | 1.34 | 22 | 266 | 0.1434 |
| Two-way effects | 18.63 | 34 | 254 | <0.001 |  | 5.34 | 34 | 254 | <0.001 |  | 10.62 | 34 | 254 | <0.001 |
|  | **CRPA** | | | |  | **3GCREC** | | | |  | **3GCRKP** | | | |
| Individual effects | 39.22 | 12 | 276 | <0.001 |  | 71.12 | 12 | 276 | <0.001 |  | 21.46 | 12 | 276 | <0.001 |
| Time effects | 0.87 | 22 | 266 | 0.6295 |  | 3.7 | 22 | 266 | <0.001 |  | 1.35 | 22 | 266 | 0.1393 |
| Two-way effects | 14.21 | 34 | 54 | <0.001 |  | 28.07 | 34 | 254 | <0.001 |  | 8.8 | 34 | 254 | <0.001 |

**Table S9 Specification test for panel models.**

| Hausman test | Fe-Re | |
| --- | --- | --- |
|  | **χ2** | p |
| **CRAB** | 53.79 | <0.001 |
| **CREC** | 28.64 | 0.0014 |
| **CRKP** | 93.17 | <0.001 |
| **CRPA** | 57.53 | <0.001 |
| **3GCREC** | 45.44 | <0.001 |
| **3GCRKP** | 31.65 | 0.0005 |

|  | **CRAB** | |  | **CREC** | |  | **CRKP** | |  | **CRPA** | |  | **3GCREC** | |  | **3GCRKP** | |
| --- | --- | --- | --- | --- | --- | --- | --- | --- | --- | --- | --- | --- | --- | --- | --- | --- | --- |
|  | *Coefficient [95%CI]* | *P-value* |  | *Coefficient [95%CI]* | *P-value* |  | *Coefficient [95%CI]* | *P-value* |  | *Coefficient [95%CI]* | *P-value* |  | *Coefficient [95%CI]* | *P-value* |  | *Coefficient [95%CI]* | *P-value* |
| Tmp | -0.093  [-0.221, 0.036] | 0.141 |  | 0.206  [0.018, 0.394] | 0.035 |  | -0.104  [-0.280, 0.071] | 0.219 |  | -0.002  [-0.138, 0.134] | 0.972 |  | 0.093  [-0.006, 0.192] | 0.063 |  | -0.097  [-0.257, 0.064] | 0.214 |
| log_PREC | -0.047  [-0.337, 0.243] | 0.730 |  | -0.033  [-0.467, 0.402] | 0.873 |  | -0.217  [-0.651, 0.218] | 0.298 |  | 0.164  [-0.080, 0.408] | 0.169 |  | 0.091  [-0.216, 0.399] | 0.531 |  | 0.325  [-0.013, 0.662] | 0.058 |
| log_PM2.5 | 0.159  [-0.214, 0.531] | 0.372 |  | -0.489  [-1.021, 0.042] | 0.068 |  | -0.030  [-0.632, 0.572] | 0.915 |  | 0.202  [-0.108, 0.513] | 0.180 |  | 0.112  [-0.317, 0.542] | 0.580 |  | 0.501  [-0.045, 1.047] | 0.069 |
| log_PD | 1.108  [-0.732, 2.949] | 0.214 |  | -1.971  [-4.795, 0.854] | 0.154 |  | 0.689  [-2.158, 3.535] | 0.608 |  | 0.985  [-0.223, 2.192] | 0.101 |  | 1.341  [-0.184, 2.866] | 0.079 |  | 0.438  [-1.114, 1.989] | 0.550 |
| log_HD | 0.318  [-0.031, 0.666] | 0.070 |  | -0.105  [-0.634, 0.423] | 0.672 |  | 0.112  [-0.345, 0.569] | 0.603 |  | -0.106  [-0.448, 0.235] | 0.510 |  | -0.095  [-0.303, 0.114] | 0.343 |  | -0.296  [-0.498, -0.093] | 0.008 |
| HEG | -0.114  [-0.244, 0.017] | 0.081 |  | 0.031  [-0.118, 0.180] | 0.658 |  | -0.075  [-0.267, 0.118] | 0.414 |  | 0.016  [-0.061, 0.093] | 0.658 |  | -0.004  [-0.063, 0.054] | 0.874 |  | 0.064  [0.026, 0.102] | 0.003 |
| UP | -0.006  [-0.056, 0.044] | 0.803 |  | -0.003  [-0.074, 0.068] | 0.933 |  | 0.024  [-0.055, 0.104] | 0.523 |  | -0.010  [-0.037, 0.167] | 0.418 |  | 0.024  [0.004, 0.045] | 0.026 |  | -0.006  [-0.044, 0.032] | 0.719 |
| CPI | 0.008  [-0.006, 0.021] | 0.251 |  | 0.006  [-0.022, 0.034] | 0.643 |  | -0.001  [-0.025, 0.023] | 0.943 |  | 0.001  [-0.012, 0.015] | 0.843 |  | 0.000  [-0.020, 0.020] | 0.992 |  | 0.017  [0.001, 0.033] | 0.035 |
| AMC | 0.020  [0.006, 0.035] | 0.010 |  | 0.017  [-0.047, 0.080] | 0.579 |  | 0.041  [0.001, 0.081] | 0.047 |  | 0.014  [0.004, 0.023] | 0.008 |  | 0.001  [-0.036, 0.038] | 0.940 |  | -0.033  [-0.075, 0.008] | 0.106 |
| WASH | -0.029  [-0.065, 0.006] | 0.100 |  | 0.069  [0.005, 0.132] | 0.036 |  | -0.011  [-0.059, 0.037] | 0.632 |  | 0.029  [0.007, 0.050] | 0.014 |  | 0.009  [-0.012, 0.030] | 0.367 |  | -0.043  [-0.068, -0.018] | 0.003 |
| R2_within | 0.40 |  |  | 0.12 |  |  | 0.28 |  |  | 0.33 |  |  | 0.47 |  |  | 0.37 |  |
| N | 299 |  |  | 299 |  |  | 299 |  |  | 299 |  |  | 299 |  |  | 299 |  |

**Table S10 Results of the fixed individual effects**

|  | **CRAB** | |  | **CREC** | |  | **CRKP** | |  | **CRPA** | |  | **3GCREC** | |  | **3GCRKP** | |
| --- | --- | --- | --- | --- | --- | --- | --- | --- | --- | --- | --- | --- | --- | --- | --- | --- | --- |
|  | *Coefficient [95%CI]* | *P-value* |  | *Coefficient [95%CI]* | *P-value* |  | *Coefficient [95%CI]* | *P-value* |  | *Coefficient [95%CI]* | *P-value* |  | *Coefficient [95%CI]* | *P-value* |  | *Coefficient [95%CI]* | *P-value* |
| Tmp | -0.044  [-0.119, 0.030] | 0.218 |  | 0.155  [-0.099. 0.409] | 0.207 |  | -0.084  [-0.347, 0.180] | 0.501 |  | -0.046  [-0.132, 0.040] | 0.267 |  | -0.023  [-0.149, 0.104] | 0.705 |  | -0.007  [-0.137, 0.123] | 0.907 |
| log_PREC | 0.087  [-0.210, 0.384] | 0.536 |  | -0.074  [-0.358, 0.211] | 0.583 |  | 0.194  [-0.347, 0.736] | 0.449 |  | 0.201  [-0.075, 0.478] | 0.139 |  | 0.065  [-0.167, 0.298] | 0.551 |  | 0.151  [-0.209, 0.510] | 0.379 |
| log_PM2.5 | 0.105  [-0.367, 0.576] | 0.637 |  | -0.611  [-1.271, 0.050] | 0.067 |  | 0.176  [-0.551, 0.903] | 0.608 |  | 0.383  [-0.086, 0.852] | 0.100 |  | 0.264  [-0.206, 0.733] | 0.245 |  | 0.365  [-0.122, 0.853] | 0.129 |
| log_PD | 3.514  [0.228, 6.799] | 0.038 |  | -3.799  [-12.493, 4.895] | 0.360 |  | 5.717  [2.555, 8.879] | 0.002 |  | 1.088  [-0.528, 2.703] | 0.168 |  | -0.497  [-3.612, 2.618] | 0.734 |  | 0.413  [-3.521, 4.347] | 0.823 |
| log_HD | 0.333  [-0.037, 0.703] | 0.073 |  | -0.087  [-0.708, 0.533] | 0.764 |  | 0.105  [-0.218, 0.428] | 0.493 |  | -0.108  [-0.463, 0.247] | 0.520 |  | -0.103  [-0.292, 0.085] | 0.256 |  | -0.345  [-0.554, -0.136] | 0.004 |
| HEG | -0.047  [-0.171, 0.077] | 0.424 |  | -0.036  [-0.218, 0.146] | 0.672 |  | 0.077  [-0.088, 0.242] | 0.327 |  | 0.020  [-0.097, 0.138] | 0.712 |  | -0.059  [-0.161, 0.043] | 0.232 |  | 0.096  [-0.013, 0.205] | 0.078 |
| UP | 0.016  [-0.033, 0.065] | 0.485 |  | -0.020  [-0.151, 0.110] | 0.737 |  | 0.070  [0.007, 0.133] | 0.032 |  | -0.011  [-0.044, 0.022] | 0.479 |  | 0.006  [-0.038, 0.050] | 0.771 |  | -0.004  [-0.061, 0.054] | 0.896 |
| CPI | 0.011  [-0.002, 0.024] | 0.090 |  | 0.004  [-0.024. 0.032] | 0.775 |  | 0.005  [-0.012, 0.022] | 0.520 |  | 0.001  [-0.137, 0.017] | 0.838 |  | -0.003  [-0.023, 0.018] | 0.779 |  | 0.019  [0.003, 0.034] | 0.026 |
| AMC | 0.023  [0.001, 0.045] | 0.040 |  | 0.018  [-0.054. 0.090] | 0.591 |  | 0.045  [0.028, 0.061] | <0.001 |  | 0.013  [0.004, 0.022] | 0.008 |  | 0.000  [-0.036, 0.035] | 0.980 |  | -0.038  [-0.074, -0.003] | 0.037 |
| WASH | -0.026  [-0.065, 0.013] | 0.177 |  | 0.065  [-0.002. 0.131] | 0.057 |  | -0.009  [-0.052, 0.035] | 0.666 |  | 0.024  [0.003, 0.046] | 0.027 |  | 0.002  [-0.019, 0.023] | 0.806 |  | -0.030  [-0.055, -0.005] | 0.024 |
| R2_within | 0.51 |  |  | 0.15 |  |  | 0.51 |  |  | 0.37 |  |  | 0.55 |  |  | 0.44 |  |
| N | 299 |  |  | 299 |  |  | 299 |  |  | 299 |  |  | 299 |  |  | 299 |  |

**Table S11 Results of the two-way fixed effects model**

1. [WHO Western Pacific | World Health Organization](https://www.who.int/westernpacific/) [↑](#footnote-ref-0)
