# Supplementary material for: Climate change and antimicrobial resistance in the Western Pacific: a mixed-methods systematic analysis
Source: Lancet Reg Health West Pac. 2025 Dec 16;67:101772. doi: 10.1016/j.lanwpc.2025.101772 (PMC12768945; doi:10.1016/j.lanwpc.2025.101772)
Supplement: Abstract in Chinese [file mmc2.docx]

***Editor disclaimer:***

*This translation in Chinese was submitted by the authors and we reproduce it as supplied. It has not been peer reviewed. Our editorial processes have only been applied to the original abstract in English, which should serve as reference for this manuscript.*

**背景**

气候变化与抗菌药物耐药性（AMR）是全球范围内日益严峻的公共卫生威胁。西太平洋地区面临着独特的气候和社会经济脆弱性，但关于气候与AMR交叉领域的研究证据有限。本研究旨在为这一关键问题系统性地提供证据。

**方法**

我们进行了一项三阶段混合方法系统分析：(1) 一项叙述性综述，用于描绘区域AMR格局并总结潜在的气候驱动机制； (2) 一项针对区域内定量研究的系统综述（检索PubMed和Google Scholar，2000年1月至2025年3月）； (3) 一项使用纵向面板数据的实证定量分析。该分析通过可视化AMR死亡率趋势（使用GRAM项目数据）并应用回归分析，基于气候和社会经济因素对AMR所致的死亡率进行建模，从而为区域现状及其潜在驱动因素提供了定量证据，并以此构成了我们完整的系统性分析。

**结果**

文献综述证据表明，气候变化导致的温度升高会直接加速细菌生长和耐药突变率，并间接导致极端天气事件期间的医疗服务中断和抗生素滥用。我们纳入了18项定量研究，并使用SWiM框架进行综合，提供了更具体的证据，表明温度升高与临床耐药率增加以及抗菌药物耐药基因（ARGs）在环境中的传播加剧有关。我们的定量分析发现，平均环境温度每升高1°C，碳青霉烯类耐药鲍曼不动杆菌（CRAB）所致的死亡率随之增加（β=0.652, 95% CI 0.579–0.724, p<0.001），碳青霉烯类耐药铜绿假单胞菌（CRPA）所致的死亡率亦随之增加（β=0.422, 95% CI 0.304–0.541, p<0.001）。分析还揭示了社会经济因素具有异质性影响。

**阐释**

气候条件和社会经济脆弱性共同塑造了西太平洋地区的AMR风险。预计极端天气事件的增加将进一步使医疗系统承压，并加剧抗生素滥用。加强气候韧性卫生系统、改善AMR多部门治理以及建立一体化AMR-气候监测网络是该地区的重要优先事项。

**关键词**

气候变化; 抗菌药物耐药性; 气候韧性卫生系统; 脆弱性
